# Phenotypic Plasticity in Circulating Tumor Cells Is Associated with Poor Response to Therapy in Metastatic Breast Cancer Patients

**DOI:** 10.3390/cancers15051616

**Published:** 2023-03-06

**Authors:** Evan N. Cohen, Gitanjali Jayachandran, Hui Gao, Phillip Peabody, Heather B. McBride, Franklin D. Alvarez, Megumi Kai, Juhee Song, Yu Shen, Jie S. Willey, Bora Lim, Vicente Valero, Naoto T. Ueno, James M. Reuben

**Affiliations:** 1 Morgan Welch Inflammatory Breast Cancer Research Program and Clinic, The University of Texas MD Anderson Cancer Center, Houston, TX 77030, USA; 2 Department of Hematopathology Research, Division of Pathology and Laboratory Medicine, The University of Texas MD Anderson Cancer Center, Houston, TX 77030, USA; 3 Department of Breast Medical Oncology, The University of Texas MD Anderson Cancer Center, Houston, TX 77030, USA; 4 Department of Biostatistics, The University of Texas MD Anderson Cancer Center, Houston, TX 77030, USA; 5 Breast Cancer Research Program, Baylor College of Medicine, Houston, TX 77030, USA; 6 Cancer Biology and Therapeutic Program, University of Hawai’i Cancer Center, Honolulu, HI 96822, USA

**Keywords:** circulating tumor cells (CTCs), neoplastic cells, circulating, neoplasms/diagnosis, circulating/pathology, biopsy, breast neoplasms/pathology, breast cancer, biomarkers, tumor, blood, liquid biopsy, metastatic process, EMT

## Abstract

**Simple Summary:**

Circulating tumor cells (CTCs) have served as an independent prognostic factor in the management of metastatic breast cancer (MBC). Through the enrichment of CTCs from peripheral blood, tumor cells can be acquired multiple times during therapy and provide a broad sample of tumor heterogeneity, thereby offering a complementary approach to tissue biopsy. Traditionally, CTCs have been enriched from blood based on the expression of epithelial-specific surface proteins. However, this approach might miss the migratory cells that lack epithelial features and favor the expression of more mesenchymal features. Therefore, enrichment of CTCs based on size and deformability may capture a wider range of tumor cells in circulation. Here we present a longitudinal study using a novel microcavity array to enrich CTCs and find that a shift from epithelial CTCs to those with a mesenchymal expression pattern is associated with inferior clinical outcomes.

**Abstract:**

Circulating tumor cells (CTCs) are indicators of metastatic spread and progression. In a longitudinal, single-center trial of patients with metastatic breast cancer starting a new line of treatment, a microcavity array was used to enrich CTCs from 184 patients at up to 9 timepoints at 3-month intervals. CTCs were analyzed in parallel samples from the same blood draw by imaging and by gene expression profiling to capture CTC phenotypic plasticity. Enumeration of CTCs by image analysis relying primarily on epithelial markers from samples obtained before therapy or at 3-month follow-up identified the patients at the highest risk of progression. CTC counts decreased with therapy, and progressors had higher CTC counts than non-progressors. CTC count was prognostic primarily at the start of therapy in univariate and multivariate analyses but had less prognostic utility at 6 months to 1 year later. In contrast, gene expression, including both epithelial and mesenchymal markers, identified high-risk patients after 6–9 months of treatment, and progressors had a shift towards mesenchymal CTC gene expression on therapy. Cross-sectional analysis showed higher CTC-related gene expression in progressors 6–15 months after baseline. Furthermore, patients with higher CTC counts and CTC gene expression experienced more progression events. Longitudinal time-dependent multivariate analysis indicated that CTC count, triple-negative status, and CTC expression of *FGFR1* significantly correlated with inferior progression-free survival while CTC count and triple-negative status correlated with inferior overall survival. This highlights the utility of protein-agnostic CTC enrichment and multimodality analysis to capture the heterogeneity of CTCs.

## 1. Introduction

Metastatic breast cancer (MBC) was the leading cause of death in women worldwide in 2018 WHO World Cancer Report, 2020–2021, https://iarc.who.int/biennial-report-2020–2021web/, accessed on 2 March 2023). The high mortality of MBC could be attributed to the extraordinary tenacity of cells that travel through the bloodstream and house themselves in conducive locations, generating distant metastasis. The voyage of circulating tumor cells (CTCs) through blood is a complex biological phenomenon that is still being unraveled. Not all CTCs survive the harsh blood environment to be successfully housed in a metastatic site [1]. Hence, these highly tenacious cells carry the molecular profile that plays a crucial role in metastasis, and understanding it not only sheds light on the disease biology but also provides a more clinically valid prognosis for the patient. CTCs also carry the potential for ex vivo exploration of therapeutic agents, especially the identification of actionable targets in a companion diagnostic setting for cancers [2]. Also, molecular characteristics of CTCs could reflect intratumor heterogeneity and may explain the discrepancy often seen in gene expression patterns between primary tumors and CTCs [3].

For a cancer cell to intravasate from the initial tumor site [4], survive in the blood, evade host immune defenses, and reestablish tumor growth in a pre-metastatic niche [5], it must maintain the ability to express several different phenotypic programs [6]. This CTC plasticity affects invasion, survival, and proliferation and to a certain extent is mirrored by the typical heterogeneity of cancer [7,8]. For example, established tumors are primarily epithelial while the migratory cells show mesenchymal features. Cells from a primary tumor must undergo epithelial-to-mesenchymal transition (EMT) as an early step in the metastatic cascade to enter the bloodstream. Multiple hybrid epithelial/mesenchymal phenotypes situated along an EMT spectrum account for intra-patient temporal and spatial heterogeneity. Furthermore, the acquisition of EMT is often associated with features that define cancer stem cells (CSCs)—namely, the enhanced potential for self-renewal, tumor initiation, invasiveness, motility, and heightened resistance to apoptosis—and is also instrumental for metastasis, suggesting that a subset of CTCs with high metastatic potential might be CSCs [9]. Since CSCs are often associated with EMT [10], it is critical to capture CTCs with mesenchymal features. However, the gold standard, FDA-cleared CELSEARCH platform enumerates EpCAM-expressing CTC and may not enrich cells in EMT [11]. Furthermore, the inter- as well as intra-tumor heterogeneity of antigen expression may limit the efficiency of single or even multiantigen immunoaffinity enrichment [12,13]. Therefore a label-free enrichment method may capture a broader sample of the CTC population.

To test the relevance of the temporal heterogeneity of CTCs undergoing EMT in patient prognosis in MBC, we enriched CTCs using a microcavity array platform [14,15,16] that employs a biophysical strategy that is agnostic to cell surface protein expression and thereby can capture a wide variety of CTCs including cells with mesenchymal features. Two parameters, namely, enumeration and gene expression profiles, were used to explore the larger picture of prognostic implications of CTCs in a large clinical trial of MBC patients followed using longitudinal peripheral blood samples for up to 2 years after initiation of therapy. Temporal bulk gene expression profiles of the CTCs from these patients served as a surrogate for the phenotypic plasticity and heterogeneity that help drive metastasis and disease progression. This clinical study further demonstrates that CTC enumeration and gene expression analysis complement each other as liquid biopsy tools.

## 2. Materials and Methods

### 2.1. Patients and Healthy Volunteers

This was a single-institution study designed to prospectively collect blood samples from patients with any subtype of newly diagnosed MBC at The University of Texas MD Anderson Cancer Center. For hormone receptor–positive (HR+) MBC, enrollment was allowed in patients undergoing first-line, second-line, or third-line treatment for MBC, while for HR-negative MBC, only patients undergoing first-line therapy for MBC were enrolled. In both cases, previous treatment for the pre-metastatic disease was allowed. Patients with metastatic disease of the brain, leptomeningeal disease, or concurrent malignancies were excluded. As there is a range of MBC subtypes, patients were treated with a range of therapeutic regimens. The clinical trial was designed to enroll 200 patients and obtain a baseline sample and up to 8 longitudinal follow-up samples for a total of up to 9 samples per patient. Patients were able to remain in the study regardless of the therapy used (standard of care or clinical trials and any changes in therapy) or disease progression during the study. All patients had blood drawn every 3 months (±1 month) during their routine blood draws per their treatment schedule and roughly concurrent with their imaging tests for 2 years, as available. The first baseline MBC patient sample was processed on 12 October 2016, and samples accrued until 31 December 2020, were processed and analyzed for this study. All study participants and healthy donors (HDs) provided written informed consent and were recruited under protocols approved by the Institutional Review Board (IRB) of MD Anderson Cancer Center. A total of 184 patients provided baseline and follow-up samples, totaling 733 samples that were collected according to IRB-approved protocol PA16–0507. HDs provided 118 blood samples for this study under IRB-approved protocol PA14–0063. Each HD self-reported being cancer free at the time of the study. The study was conducted in accordance with the Declaration of Helsinki. Study outcomes did not affect the clinical management of the enrolled patients.

### 2.2. Workflow of Sample Processing

Two tubes of peripheral blood with EDTA anticoagulant were acquired from each patient at each timepoint with ~9.5 mL collected per tube. The two tubes were subjected to parallel CTC enhancement procedures, one for enumeration and the second for molecular characterization of the enriched CTCs. CTCs were agnostically enriched with a microcavity array (MCA) from Hitachi Chemical Co. (now Showa Denko Materials Co., Tokyo, Japan) [15].

#### 2.2.1. Sample Processing for Enumeration of Captured CTCs

For CTC enumeration, CTC-enriched fractions were stained in situ within the capture chip with a cocktail of antibodies and DAPI prior to image analysis. Chips were typically imaged within 2 days of the blood draw by confocal microscopy (Flow Cytometry and Cellular Imaging Core Facility at MD Anderson). CTCs were identified as pan-cytokeratin (CK)+ CD45− nucleated cells (DAPI+) based on guidelines published by Zeune and colleagues [17].

#### 2.2.2. Sample Processing Workflow for Gene Expression Analysis of Captured CTCs

Leukocytes were depleted prior to CTC enrichment and the lysed CTCs were archived in Trizol (Thermo Fisher Scientific, Waltham, MA, USA) at −80 °C until further use. HD and patient samples were subjected to gene expression analysis by quantitative real-time polymerase chain reaction (qRT-PCR) in batches at the end of the study. HD samples were randomized with the patient samples and tested concurrently. The Minimum Information for Publication of Quantitative Real-Time PCR Experiments (MIQE) guidelines was applied in the design of the primers [18] and wet lab validated by the manufacturer. A Qiagility liquid handler (Qiagen) was employed to set up PCR reactions. Additional details have been previously published [16].

For gene expression, custom-designed 384-well plates, including CTC-related genes, housekeeping control genes, and hematopoietic control genes were evaluated by qRT-PCR to characterize cells of epithelial, mesenchymal, and cancer stem cell lineage. The CTC panels included genes related to epithelial characteristics (*CDH1*, *EGFR*, *EPCAM*, *KRT7*, *KRT18*, *MUC1*), epithelial to mesenchymal transition (EMT) characteristics (*AXL*, *CDH2, FN1*, *SNAI2, ZEB2*), cancer stem-like lineage (*ALDH1A1*), and signaling pathways commonly perturbed in cancer (*BCL2*, *CD274/PD-L1*, *ERBB2*, *FGFR1*, and *MET*). Control genes included *PTPRC* (*CD45*) as a white blood cell control, *GYPA* as a nucleated red blood cell control, and housekeeping genes *B2M*, *GAPDH* and *HPRT1* as positive controls (Table S1).

From 184 patients (enrolled October 2016 through December 2020), 733 samples were subjected to gene expression analysis. Only 2 patients missed baseline samples. HD blood (118 samples) processed by MCA was used to establish a threshold for positive gene expression. Additionally, thresholds were set separately for samples processed with or without RBC (red blood cell) lysis based on the gene expression levels of 54 HD samples processed with RBC lysis and 55 without RBC lysis. A gene was considered positive if its expression was higher than 1 standard deviation above its mean expression in samples from HDs.

#### 2.2.3. EM Score

An epithelial-mesenchymal score (EM score) was calculated based on the total expression of the target genes, resulting in a score of +1 if only mesenchymal genes and stem cell–related genes were detected and a score of –1 if only epithelial genes were detected. For the EM score, *CDH1*, *EPCAM*, *KRT7*, and *KRT18* were considered epithelial (*epi*) while *ALDH1A1*, *CDH2*, *FN1*, and *ZEB2* (*mes*) were considered mesenchymal. Genes with ambiguous epithelial/mesenchymal polarity characteristics such as *FGFR1* were not considered here.

$$EM\;Score=\frac{\sum_{mes}^{4}\left(40-Ct_{mes}\right)-\sum_{epi}^{4}\left(40-Ct_{epi}\right)}{\sum_{all}^{8}\left(40-Ct_{all}\right)}$$

### 2.3. Statistical Analysis

CTC count and gene expression levels were measured at baseline and then at 2- to 3-month intervals, thereafter. The effect of baseline CTC count (<5 vs. ≥5 CTCs) and baseline gene expression (low: ≤mean +1 standard deviation of HD level vs. high: >mean +1 standard deviation) on progression and death were evaluated using Cox proportional hazards models. Time to progression and time to death was calculated from the study enrollment consent date. Patients who died without indication of progression were considered to have experienced progression on the date of death. The temporal effect of the CTC count (<5 vs. ≥5 CTCs) and gene expression groups (≤mean + 1SD of healthy donor controls as low vs. >mean + 1SD as high) on progression and the effect on death were evaluated using Cox proportional hazards models with time-dependent covariates. CTC counts greater than 40 were truncated as 40. For recurrent outcomes of progression events, the Prentice, Williams, and Peterson counting process model [19] and a sandwich estimate of the variance-covariance matrix was used to obtain standard errors accommodating the clustering of observations on patients. The last-value-carried-forward method was used to assign CTC count and gene expression levels for each patient and time interval. Incident rates (progression rate and death rate) by subgroup were calculated as the number of events per 100 person-years, with 95% confidence intervals. Mean cumulative number of progression events according to baseline CTC count (≥5 vs. <5 CTCs) was estimated using the Nelson estimator [20]. Analyses were conducted with SAS (9.4 SAS Institute INC, Cary, NC, USA) with figures generated with R version 4.1 (R Project for Statistical Computing, Vienna, Austria), using packages tidyr, survminer, survivalAnalysis, complexHeatmap, ggpubr, and swimplot.

## 3. Results

### 3.1. Patient Characteristics

One hundred eighty-four (184) patients were enrolled following a new diagnosis of MBC prior to front-line therapy for current metastatic disease. Many had previously been treated for non-metastatic disease; however, 45 patients had de novo stage IV disease. During follow-up, 80 patients died, and 122 patients had disease progression (including 17 who died without indication of progression). Fourteen patients were reported disease free (no evidence of disease, NED) at the last follow-up. The median follow-up was 24.6 months (95% CI, 21.4–30.4 months) and the median overall survival (OS) was 43.6 months (95% CI, 33.4 months–not estimated). The median time to first progression (or death) was 10.4 months (95% CI, 8.1–15.3 months). Among 184 patients, the best responses included 7 patients with complete response, 58 with partial response, 50 with stable disease, 43 with progressive disease, and 26 not evaluated. Therefore, 115 patients had some clinical benefit from therapy. There was a range of breast cancer subtypes based on pathological analysis of metastatic tumors, but HER2+ disease was slightly under-represented with 24 patients (Table 1). The number of patients that provided samples at each timepoint is displayed in the study design summary (Figure S1).

### 3.2. Enumeration of CTCs

#### 3.2.1. Longitudinal Distribution of CTC Counts during Therapy

**Figure 1:**
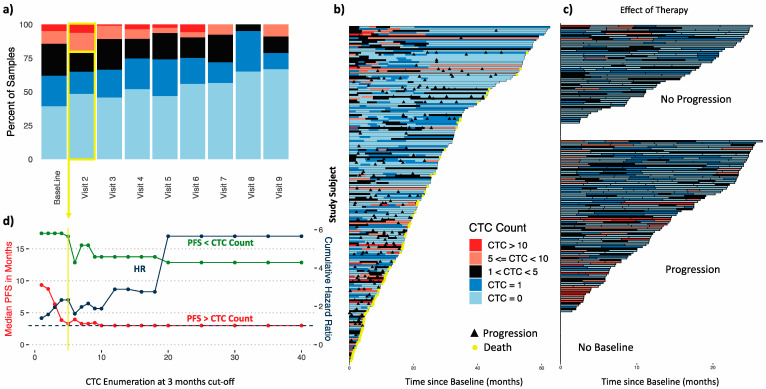
CTC count distribution. (**a**) Range of CTC counts per ~9.5 mL of blood across time points. (**b**) Swimmer plots of CTC counts demonstrate that enumeration is prognostic. (**c**) Swimmer plots of CTC count from cases that progressed and those that did not progress demonstrate the effect of therapy. (**d**) CTC enumeration at 3 months confirms ≥5 CTCs/9.5 mL of blood as the most relevant cutoff. Median PFS for patients above (green) and below (red) each CTC threshold (left y axis) and associated hazard ratio (blue, right y axis, dashed blue line HR = 1). CTC, circulating tumor cell; HR, hazard ratio; PFS, progression-free survival.

CTCs from patients with MBC were enumerated in real-time at the time of image acquisition. Across timepoints, 84% of the MBC patients had detectable CTCs from at least one blood draw over the course of the study. At baseline, 39% of patients had 0 CTCs, about 62% had 0 or 1 CTCs (≤1 CTC), and 84% had fewer than 5 CTCs (Figure 1a). The highest CTC count observed was about 5000 CTCs (visit 5). Enumerated CTCs generally decreased as time-on-study progressed. Median CTC counts were significantly lower than baseline from visit 4 (9 months) onward (Wilcoxon rank sum test *p* < 0.05, Figure S2). Archived images were subsequently analyzed by an independent observer as well as image analysis software, showing similar results (not shown).

Patients who experienced progression at some point while on protocol had significantly higher CTC counts at baseline and 3 months (visit 2) (Figure S3a,b, Wilcoxon rank sum *p* < 0.05).

The CTC counts and progression events for each patient at each timepoint are represented in a swimmer plot (Figure 1b). Looking at the effect of therapy (with no consideration of baseline measurement), several clear distinctions can be seen in CTC counts between patients who experienced disease progression and those who did not (Figure 1c). CTC counts ≥1 were concentrated in patients with the shortest study times. In contrast, patients without disease progression (Figure 1c, top) have generally low CTC counts (shades of blue). Several of these patients had high counts at earlier timepoints that decreased by later timepoints, suggesting a positive response to therapy. These results highlight the utility of CTC enumeration in patients with MBC, as has been extensively documented.

#### 3.2.2. Established CTC Threshold

Prior to further analysis, we first determined the optimal cutoff for CTC enumeration to stratify patients. Univariate Cox regression was used to analyze the effect of CTC enumeration using different cutoffs at first follow-up (3 months) on time to first progression (progression-free survival, PFS) including all 184 patients with MBC (Figure 1d) reaffirming ≥5 CTCs as the optimal cutoff with remarkably similar characteristics to the original FDA submission by CELLSEARCH. A similar cutoff was obtained with a similar analysis of the baseline blood sample or all samples combined (Figure S4).

#### 3.2.3. CTC Enumeration Predicts Survival

**Figure 2:**
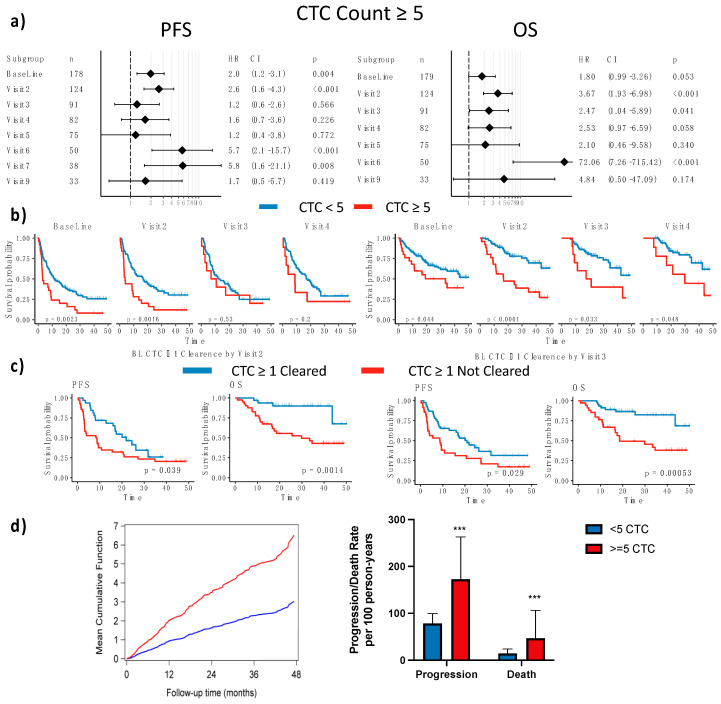
CTC count ≥5 is prognostic at the start of therapy for MBC. (**a**) Forest plots showing Cox proportional hazard for ≥5 CTCs/~9.5 mL of blood at each timepoint for PFS and OS. (**b**) Kaplan-Meier plots for PFS and OS stratified by ≥5 CTCs ~9.5 mL of blood. (**c**) Kaplan-Meier plots stratified by clearance of ≥5 baseline CTCs by visits 2 and 3. (**d**) Mean cumulative progression events for ≥5 CTCs vs. <5 CTCs at baseline and progression and death rates per 100 person-years. CTC, circulating tumor cell; OS, overall survival; PFS, progression-free survival. *** *p* < 0.0001.

Using the cutoff of ≥5 CTCs per blood sample, established previously for other platforms and reaffirmed above, CTC counts were prognostic in patients with MBC starting a new therapy. Cox proportional hazard modeling showed that CTC counts were prognostic for PFS before therapy (baseline) and at early points in therapy (visit 2, about 3 months) (baseline HR for first progression = 1.97, *p* = 0.004; visit 2 HR for first progression = 2.6, *p* < 0.001). However, CTC counts were not significant prognostic factors of progression for the remainder of the first year (visits 3, 4, and 5). However, CTC count regained significance after the first year of therapy (visit 6 (15 months) and visit 7 (1.5 years)), when there is a likely survivor bias with an early drop out of patients whose therapy failed to clear high levels of CTCs (Figure 2a,b). Similarly, there was a trend for CTC counts to predict OS at baseline and significant effects early in therapy (visit 2 (3 months), visit 3 (6 months)), (baseline HR for first progression = 1.78, *p* = 0.053; visit 2 HR for first progression = 3.67, *p* < 0.001; visit 3 HR for first progression = 2.47, *p* = 0.041), with trends continuing at later timepoints.

In a multivariate Cox analysis that included age and tumor subtype (as determined from a biopsy of metastatic tumor tissue), a CTC count of ≥5 CTCs remained an independent prognostic factor for PFS and OS at 3 months, 15 months, and 18 months but only for PFS at baseline (Figure S5). Repeated CTC enumeration using archived images by an independent observer, while not independent of the original count, was also an independent prognostic factor when substituted for the initial count (for example, in the baseline model of PFS, HR = 2.58, *p* < 0.001 for the independent repeated count).

#### 3.2.4. Dynamics of CTC Counts during Therapy

High CTC counts can predict progression before definitive manifestation by clinical imaging. To quantify the lead time provided by high CTC counts, we measured the time to first progression in patients with ≥5 CTCs. There were 28 patients with ≥5 CTCs at baseline, of whom 25 progressed with a median time to progression of 2.99 months. Several patients had disease progression at or after the second visit; of the 14 patients with ≥5 CTCs at the second visit who subsequently progressed, the average time to documented clinical progression by imaging was 6.34 months after ≥5 CTCs were detected. A similar pattern emerged at 6 months (visit 3) with 20.1 months lead time, albeit with only 3 patients with CTC ≥5.

Since reductions in CTC counts, representing clearance of CTC during therapy, may be indicative of therapeutic response, we looked at changes in CTC counts over time. Dynamic longitudinal changes in CTC status with a cutoff of ≥5 CTCs offered similar prognostic value as static evaluation: decreases in CTC counts from baseline were significantly associated with improved prognosis only at the 3-month visit, but not at subsequent follow-ups (Figure S6). Patients with any CTCs detected (>0) that were cleared by therapy had good OS (Figure 2c), but clearance was not indicative of PFS (Figure 2c), and, anecdotally, the 5 patients who had ≥5 CTCs at baseline that resolved to <5 CTCs by the first follow-up (visit 2) were still alive as of this analysis.

Baseline CTC count was also prognostic of the number of progression events in longitudinal modeling of the rates of progression and death (Figure 2d). Patients with ≥5 CTCs at baseline had a higher mean cumulative number of progressions than patients with <5 CTCs, and the difference broadened with increasing follow-up time. A CTC count of ≥5 was also associated with higher rates of progression and death than CTCs <5. For progression, the incidence rate ratio (IRR) of a CTC count of ≥5 to a CTC count of <5 was significantly higher than 1 (IRR = 2.03, 95% CI, 1.53–2.69). For OS, the IRR of ≥5 CTCs to <5 CTCs was again significantly higher than 1 (IRR = 4.58, 95% CI, 2.84–7.39). Together, these data reaffirm that enumeration of CTC can help stratify patients by risk and monitor response to therapy.

### 3.3. Gene Expression in Enriched CTCs

There has been an unmet clinical need for further characterization of CTCs beyond enumeration. Since gene expression is especially relevant for deciphering EMT plasticity and also signaling pathways that are pertinent to therapeutic targets, we investigated gene expression as a prognostic factor in MBC using a pre-selected panel of CTC-related genes.

**Figure 3:**
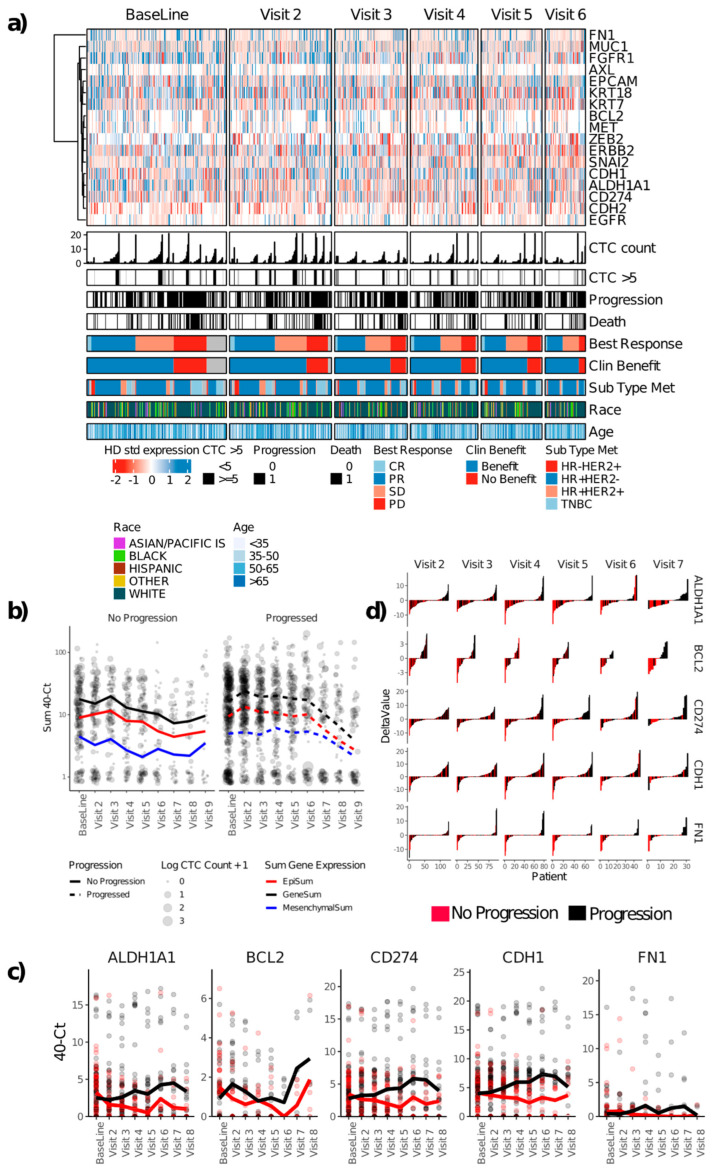
CTC gene expression. (**a**) Heat map of gene expression Z-score normalized to HD samples. (**b**) Mean sum of CTC-related gene expression (epithelial, mesenchymal, and total) stratified by progression. (**c**) Mean expression of 5 individual genes stratified by progression. (**d**) Waterfall plot showing a change in CTC-related gene expression from baseline for the same 5 genes. CTC, circulating tumor cell. CR, complete response; HR, hormone receptor (ER or PR); PD, progressive disease; PR, partial response; SD, stable disease; TNBC, triple negative (for ER, PR, and HER2).

The distribution of CTC-related gene expression is shown in Figure 3a. CTC-related genes were detected in the baseline samples of 129 patients (71%), and at least 1 cancer-related gene was detected in 486 of 677 longitudinal samples analyzed (72%). Across timepoints, 169 (93%) of the patients had at least 1 gene from the panel detectable in at least 1 sample. As with CTC count, the total expression of target genes tended to decrease with time on the protocol. As with CTC count, patients who did not experience progression on protocol had a decrease in total expression over time, including expression of both epithelial (*CDH1*, *EPCAM*, *KRT18*, *KRT7*, and *MUC1*) and mesenchymal/CSC-related genes (*ALDH1A1*, *CDH2*, *FN1*, *ZEB2*) (Figure 3b). Patients with disease progression had a significantly higher total expression of CTC-related target genes compared to patients without disease progression at visits 3, 4, 5, and 6 (Figure S7). Generally, at later timepoints, the CTC-enriched samples of patients who experienced tumor progression had higher mRNA expression of the CSC marker *ALDH1A1*, the anti-apoptotic gene *BCL2*, epithelial cell adhesion gene *CDH1* (e-cadherin), the migratory adhesion gene *FN1*, and the immune checkpoint inhibitor *CD274* (*PD-L1*) (Figure 3c). In the 9-month to 1-year range (visits 4 and 5), the CTC-enriched cells of patients with progression had higher mRNA levels of *ALDH1A1*, *AXL*, *CD274*, *CDH1*, and *ZEB2* (Wilcoxon rank sum test *p* < 0.05, Figure S8). Longitudinal changes from baseline in the expression of these genes showed similar patterns (Figure S9): Patients who did not experience progression had significant decreases from baseline in *ALDH1A1*, *BCL2*, *CD274*, *CDH1*, and *FN1*, while patients who experienced progression had significant increases in *ALDH1A1*, *BCL2*, *CD274*, and *CDH1* (Figure 3d and Figure S10). As noted above with the summed expression, there was a greater differential expression among mesenchymal genes (with the exception of *CDH1*), which highlights the benefit of a protein-agnostic enrichment approach.

**Figure 4:**
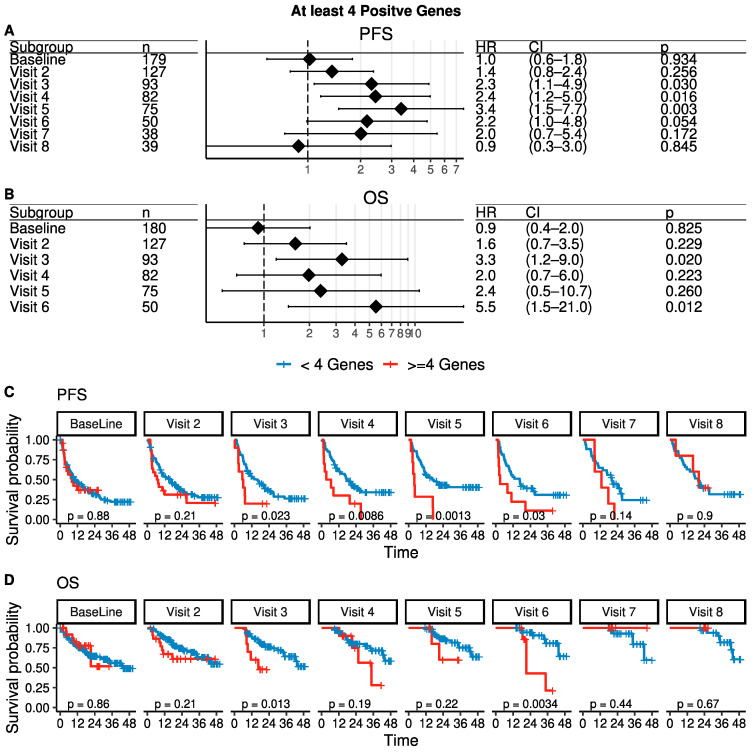
Expression of at least 4 CTC-related genes is prognostic for evaluation of response to therapy. (**A**,**B**) Forest plots showing Cox proportional hazard for PFS and OS stratified by ≥4 CTC-related genes at each timepoint. (**C**,**D**) Kaplan-Meier plots for PFS and OS stratified by ≥4 CTC-related genes. CTC, circulating tumor cell; PFS, progression-free survival; OS, overall survival.

For a better-unified summary of gene expression, we counted the number of genes that were expressed at higher levels compared to HD blood samples subjected to the same processing as patient samples using a cut-off of ≥4 positive CTC genes (Figure S11a). In contrast to CTC enumeration, ≥4 positive CTC genes was not significantly prognostic of PFS or OS at baseline nor at the first follow-up (Figure 4), although several individual genes were prognostic in univariate analysis at 3 months (Figure S11b). Conversely, and also in contrast to enumeration, gene expression of ≥4 CTC genes was associated with significantly increased risks of progression as determined by samples collected at 6 months, 9 months, and 1 year (at 1 year, HR for PFS = 3.4, *p* = 0.003, Figure 4). Interestingly, although baseline gene expression was poorly prognostic of the first progression, patients with higher baseline expression of CTC-related genes had a higher number of subsequent progression events (Figure S12).

To assess the risk associated with the detection of CTCs by either imaging or gene expression, multivariate Cox analysis including CTC count, the presence of at least 4 positive CTC genes, and the tumor subtype determined from the metastatic tumor tissue (and therefore the basis of the therapy administered at baseline in this study) was performed for each time point. As suggested above, CTCs enumerated by imaging at baseline, visit 2 at 3 months, visit 6 at 15 months, and visit 7 at 18 months were each an independent prognostic factor for PFS. In contrast to CTC enumeration, gene expression was an independent prognostic factor for PFS visit 3 at 6 months, visit 4 at 9 months, and visit 5 at 1 year (Figure S13). There were no timepoints where both CTC count and CTC gene expression were significant independent prognostic factors of PFS. In terms of OS, CTCs enumerated by imaging were an independent prognostic factor at visits 2, 3, 4, and 6. Gene expression and CTC enumeration were both independent predictors of OS only at visit 3.

### 3.4. Time-Dependent Survival Analysis

Although CTC gene expression at baseline showed marginal utility, longitudinal evaluation was a much more powerful prognostic tool. Univariate Cox regression analyses of the effects of time-dependent covariates on recurrent progression by the Prentice, Williams, and Peterson counting process model are summarized in Table S2, which shows the HR of experiencing progression for every one-unit increase in each covariate or each response of a covariate relative to a reference group. CTC count (continuous and categorical) and gene expression of CTC were considered time-dependent covariates, whereas others were baseline covariates. Covariates significantly associated with progression were number of lines of therapy, the subtype of the metastatic lesion (estrogen receptor (ER)/progesterone receptor (PR)/HER2 expression), CTC count with a threshold of ≥5 CTCs, and the CTC expression of *FGFR1*, *KRT7*, and *MUC1*; *KRT18* expression was marginally associated with increased risk of progression. This analysis shows, for example, at any given timepoint, if an MBC patient has a CTC count of ≥5, her hazard rate of progression is higher than if she had a CTC count of <5 (HR = 2.104, *p* < 0.0001).

Similarly, univariate Cox regression analyses of the effects of time-dependent covariates on death are summarized in Table S2. Covariates that were significantly associated with death were lines of therapy, primary and metastatic tumor markers (HR/HER2), CTC count ≥5, and a number of positive CTC genes. At any given timepoint, if a patient had a CTC count of ≥5, their hazard of death was higher than if they had a CTC count of <5 (HR = 4.827, *p* < 0.0001).

In multivariate Cox regression models of the effects of time-dependent covariates on progression, the covariates of metastatic tumor subtype, CTC count ≥5, and CTC expression of *FGFR1* remained significant as independent prognostic factors (Table 2 and Table S2), although in an alternate multivariate model including metastatic tumor subtype, CTC count, and *KRT7* (without *FGFR1*), *KRT7* was significant (*p* = 0.0227) but not as strong as *FGFR1* (not shown). As expected, triple-negative breast cancer, which tends to be more aggressive and lacks targeted therapy, showed an increased HR in this analysis. Notably, CTC count and an independent recount subsequently performed on archival images by an independent observer were not independent of each other, as they were highly, but imperfectly, correlated, further suggesting the CTC counts are reasonably robust (not shown). Although the amalgamated gene expression measure of any 4 positive genes was prognostic at individual timepoints (as described above in Figure 4), in the time-dependent analysis, this variable did not remain independent of CTC count and was dropped from the final model. However, a lack of independence may not imply a lack of utility. At baseline, 26 MBC patients had at least 5 CTCs. Of these, 5 had no positive genes, and only 3 had at least 5 positive genes. In contrast, 38 MBC patients had at least 5 positive genes, of whom only 3 patients had CTC counts greater than 5. Therefore, although CTC counts and CTC gene expression are correlated and are not independent prognostic factors, additional information is gained by using both measures.

At no timepoint were a CTC count ≥5 and expression of ≥4 CTC genes both significant in multivariate analysis for PFS (as shown above in Figure S13). As epithelial genes make up a large portion of the gene panel, it is intuitive that CTC count based on cytokeratin-based staining and gene expression including cytokeratins and other epithelial markers would not be independent. However, when combining all timepoints into multivariate Cox regression models of the effect of time-dependent covariates on recurrent progression, some individual genes, most prominently *FGFR1*, were independent prognostic factors along with CTC count. However, in similar models of OS, only CTC count and tumor subtype were independent prognostic factors, but no genes were included in the final model. In mixed effects modeling, *FN1*, *MUC1*, and standardized *EPCAM* were significantly associated with clinical benefit after Bonferroni-Holm correction for multiple tests (Table S3).

Overall, these results suggest that CTC enumeration and gene expression are complementary. We have shown that the enumeration of CTCs is less prognostic than gene expression while patients are in therapy. However, in a disease-monitoring setting at these intermediate timepoints, gene expression including a panel of epithelial genes, mesenchymal genes, and cancer-related genes is better able to identify patients at high risk of disease progression.

### 3.5. CTC Clusters and EMT Plasticity

**Figure 5:**
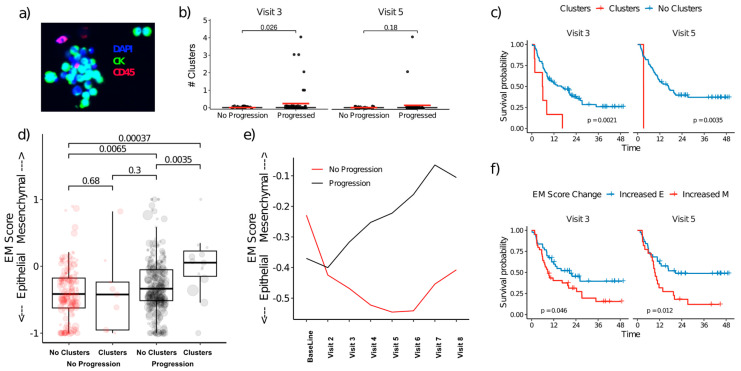
CTC clusters and EMT plasticity (**a**) Example of CTC cluster. (**b**) CTC clusters are more frequent in patients who experience progression at visits 3 (6 months) and 5 (1 year). (**c**) PFS by detection of clusters. (**d**) Median EM score stratified by patients with and without CTC clusters and in terms of their disease condition; size proportional to total CTC count. (**e**) Median EM scores across timepoints stratified by patients with and without progression where positive has greater mesenchymal gene expression and negative has greater epithelial gene expression, irrespective of the presence of clusters. (**f**) PFS by a change in EM scores from baseline. CK: pan-cytokeratin antibody; E: epithelial; M: mesenchymal; PFS, progression-free survival.

Since clusters of CTCs may have higher metastatic potential than individual cells, we compared the number of clusters observed in patients with and without progression. CTC clusters were observed in 27 samples. Between 6 months and 1 year of therapy (visits 3, 4, and 5), CTC clusters (Figure 5a) were observed only in patients who experienced progression. Furthermore, patients who experienced progression had significantly higher numbers of CTCs in clusters at 6 months (visit 3) and 1 year (visit 5) (Figure 5b), with an increased risk of disease progression (Figure 5c), and these patients exhibited an increase in the number of clusters compared to baseline. Since CTC clusters contain cells with both increased cell-cell adhesions and migratory properties that can promote metastatic seeding at distant sites, we compared the ratio of epithelial and mesenchymal gene expression (EM score) in samples with identified CTC clusters. The EM score significantly favored mesenchymal polarity in samples with CTC clusters, but only in patients with disease progression (Figure 5d). Furthermore, irrespective of the presence of clusters, CTCs from patients with disease progression had increased mesenchymal/CSC-like polarity after initiation of therapy, whereas CTCs from patients without disease progression (stable disease) shifted towards a more epithelial expression pattern (Figure 5e), with a significant difference in cohort expression patterns at the 1-year timepoint (visit 5, *t*-test *p* = 0.009). Patients with detectable CTC clusters had a greater risk of progression at 6 months (visit 3) and 1 year (visit 5) (Figure 5f). Overall, these results suggest patients with a shift towards a mesenchymal CTC phenotype, particularly within CTC clusters, are less likely to respond to therapy.

### 3.6. Selected Biomarker Examples and Case Studies

(a) HER2:

Among baseline samples, 21 MBC patients showed HER2+ CTCs, defined as ERBB2 gene expression higher than 1 standard deviation above the mean expression in the HDs. Of these, only 7 patients with MBC had HER2+ metastases by clinical evaluations (concordant with CTCs), but 11 patients with MBC had HER2− metastases, i.e., the metastatic tissue and CTC results were discordant. Of the patients with MBC with HER2-discordant CTC and metastases, 9 were evaluable for clinical benefit, of which only one received a benefit from first-line therapy for metastatic disease (Figure S14). Since these patients were HER2− by clinical evaluations, they did not receive HER2-targeted therapies. One of these patients (B152) subsequently tested positive for HER2 at a different metastatic site 4 months after the baseline CTC measurement. This patient had been placed on a HER2-targeted therapy at that time and is doing well as of this report. A second patient, B158, also subsequently tested positive for HER2. After switching to HER2-targeted therapy, this patient had a stable disease as of this report.

(b) EGFR:

Among patients with high *EGFR* expression by CTCs at baseline, only one received *EGFR*-targeted therapy and had a partial response to therapy. The other 9 patients were not treated with *EGFR*-targeted therapy; 7 of these patients have died, and 2 were alive at the last follow-up with progressive disease. Case studies have been described in the Supplementary Materials (Figures S15 and S16).

## 4. Discussion

Here, we report the results of the first longitudinal study of CTCs from patients with MBC using the protein-agnostic MCA CTC enrichment platform. In contrast to CELLSEARCH, the platform used here enriches CTCs based on size and deformability without a bias towards epithelial characteristics, potentially increasing the yield of clinically relevant CTCs. Although the MCA system enriches CTCs with both epithelial and mesenchymal features, the CELLSEARCH platform showed remarkably similar data in the initial FDA submission, and we re-affirmed the cutoff of ≥5 epithelial CTCs as the most relevant threshold for MBC prognosis.

The enumeration of CTCs by imaging had prognostic value, but with limitations. In the full patient cohort, there was a general decrease in enumerated (primarily epithelial) CTCs as the time on study progressed; median counts were significantly lower than the baseline from visit 4 (9 months) onwards (*p* < 0.05, Figure S2). Importantly, survivorship bias may account for the decreasing counts at later timepoints.

More critically, our results show that primarily epithelial CTC counts have significant prognostic utility at baseline and the first follow-up at 3 months after treatment initiation, but thereafter their utility is diminished (Figure 2). As a pure conjecture, it is possible that cytotoxic therapies distort the CTC morphology and make counts more difficult to obtain.

In MBC, dynamics of CTC counts during treatment have been repeatedly shown to be related to poor prognosis [21,22,23]. The SWOG S0500 trial failed to demonstrate that CTC enumeration could be useful in selecting new lines of therapy [24]. However, larger, more recent studies have suggested that for ER+, ERBB2− MBC, CTC count can be useful for deciding between chemotherapy and endocrine therapy [25]. Therefore, there is still debate about the clinical utility of CTC enumeration *per se*, and further characterization of CTC phenotypes can fine-tune the understanding of CTC biology.

In model systems, we have previously seen that gene expression increases with the number of CTCs [26]. Therefore, gene expression can also be a surrogate for enumerated cells. Furthermore, the increased multiplexing ability of molecular assays allows the interrogation of a broader range of phenotypes. Temporal total gene expression by our targeted panel was significantly higher in patients who developed progressive disease compared to those without progression at later timepoints but before or early in therapy (Figure S7). This is in stark contrast to CTC counts, which stratified patients by progression primarily at the earlier timepoints (Figure S3). Furthermore, as noted in multivariate survival analysis, CTC counts correlated with gene expression at some timepoints, but not at others. This may suggest that the phenotypic plasticity induced or enriched by therapy can be captured by gene expression analysis of CTC.

We observed that high expression of *CDH1*, *FGFR1*, *FN1*, *KRT7*, *KRT18*, and *MUC1* was correlated with poor prognosis. Prior to therapy, many of these genes are expected to be correlated with CTC counts. For example, the proteins for *KRT8* and *KRT7* are both targeted in the cocktail of antibodies used for image analysis, and most of the other genes were specifically chosen because they are associated with epithelial cells. CTCs that have undergone partial EMT express some mesenchymal markers and show downregulation of epithelial markers. These cells with mesenchymal features may be the population most responsible for the development of metastasis, have been related to higher stage [27] and inferior prognosis [28,29,30], and may change in response to therapy [31].

However, these CSC-like and migratory phenotypes may be both enriched and induced by therapy [32,33,34,35] or inflammation [36,37] and may be targeted by therapies such as eribulin [38]. Such cells may be more difficult to detect with imaging but are detected by analysis of the EMT-associated genes whose expression patterns may change over the course of therapy. CSCs, and by extension mesenchymal cells [10], have been shown to be resistant to therapy; however, completely mesenchymal CTCs are associated with a more favorable survival outcome in breast cancer [39]. In the current study, patients whose disease responded poorly to therapy had slightly fewer mesenchymal CTCs at the start of therapy but had a shift towards greater mesenchymal polarity during therapy (Figure 5e).

We also observed greater mesenchymal polarity in samples with CTC clusters consistent with previous reports [40]. The metastatic process is very inefficient; only a tiny portion of CTCs establish metastases [6]. However, CTCs in clusters have several advantages over single CTCs, as they may be better protected from the stresses of the bloodstream [41] and have heterogeneous cells that increase the probability of metastatic seeding [42,43,44]. Detection of CTC clusters is frequently associated with poor prognosis [45,46,47,48,49]. Clustered CTCs have been extensively shown to play a crucial role in the metastatic spread of breast cancer in advanced stages [50,51] and have increased metastatic potential compared to single CTCs [44]. More recently, clustered CTCs have been reported in early breast cancer patients [52], and are potentially more prevalent than in metastatic disease [53] highlighting the significance of liquid biopsy in cancer care. Size-based enrichment such as the one used in the current study offers a straightforward way to enrich CTC since even a two-cell cluster is significantly larger than WBC [54]. We noted that patients with progression had more CTCs in clusters after the initiation of therapy.

Intriguingly, we also observed significantly higher relative mesenchymal gene expression in patients with CTC clusters (Figure 5), with the highest mesenchymal polarity in patients with CTC clusters who experienced a disease progression. The current study is limited by the use of microscopy and gene expression analysis on parallel samples from the same blood draw such that the enumerated CTCs are not the cells subjected to EMT analysis and lacks single-cell resolution for EMT. Interestingly, it makes the correlation between clusters and EMT in parallel samples more remarkable.

A true test of CTC plasticity would require following the changes in individual cells, which is beyond the scope of this project. We attempted to monitor plasticity by interrogating CTC gene expression changes over time, with the caveat that we cannot distinguish between selection and induction. However, the data lend credence to a model of heterogeneous clusters that maintain both epithelial cell junctions with associated paracrine signaling and migratory ability affording greater plasticity and adaptation to the multiple environments encountered during the metastatic cascade. Understanding the mechanisms underlying the various forms of cell plasticity may deliver new strategies for targeting the most lethal aspects of cancer: metastasis and resistance to therapy [55].

In time-dependent multivariate analysis, expression of *FGFR1* offered the greatest independent prognostic utility among tested genes. As an expression of more traditionally epithelial genes was correlated with the CTC count in this study, it is expected that epithelial genes would not be independent of CTC count. *FGFR1* is frequently overexpressed in breast cancer [56], leading to endocrine therapy resistance [57] as well as HER2 therapy resistance [58]. Several therapies targeting *FGFR1* are currently in trials [59]. However, none of the patients enrolled began any of these therapies at baseline, so they could not be explored in this analysis. As such, the CTC expression of *FGFR1* warrants further study.

In terms of other biomarkers, the discordant HER2 expression between tumor and CTCs that we observed has been previously reported using other platforms [60,61,62,63]. HER2 expression has been shown to be highly variable within CTC [64] and may represent dynamic functional states with more rapidly growing HER2+ CTC and chemotherapy-resistant HER2- CTC [65] that can be exploited by HER2 targeting therapies [66]. Together, the EGFR and ERBB2 (HER2) gene expression data show that CTCs can be a valuable source of tumor information. The CTC data supplement the tumor tissue and may better capture tumor heterogeneity that may be prevalent in HER2+ CTC [51,52]. Of note, although numerous ERBB2 and EGFR mutations are clinically targetable, the data presented here rely on expression and not mutational status. As expression can be elevated without a mutation in the target gene, these examples may not show up on one of the now-ubiquitous cell-free DNA mutation panels.

There have been a plethora of alternative approaches that attempt to overcome the information bottleneck and extract both count and extended phenotype data from a single sample [67]. On the enrichment side, we chose a size-based, protein-agnostic enrichment method, but there are several immunocapture methods that use a panel of cell surface antibodies to capture both epithelial and mesenchymal cells. For example, Adnatest is a commercially available positive selection platform for the enrichment of CTC from breast, prostate, ovarian and colon cancer [68,69]. Among others [70], cell-surface vimentin has been proposed as a surface antigen for magnetic enrichment from squamous cell sarcoma [71], pediatric sarcoma [72], neuroblastoma [73], prostate [74], gastric [75], pancreatic [76], lung [77], and breast cancer [78]. For analysis, a higher-plex immunostain with separate cocktails for epithelial and mesenchymal markers has been used to show mesenchymal and epithelial CTC, or imaging cytof can be used to stain for multiple markers. Workflows have been established for multimodality imaging that includes morphology and fluorescence imaging [79]. Some platforms forgo enrichment altogether such as the Epic Sciences platform [80,81]. For broader characterization, many groups have published intriguing data using single-cell RNA Seq [82,83,84,85,86,87], however, the expense and low recovery are not amendable to a clinical workflow. On a narrower scale, alternative gene expression platforms such as HTG [26] can be used on fixed samples following imaging if the CTC counts are high enough or spatial gene expression platforms such as NanoString’s GeoMX.

The MCA microfluidic device employed in the study does present several limitations. Recent reports have suggested that EPCAM-negative CTC tends to be smaller and therefore may not be captured by size-based enrichment [88]. Most microfluidic devices (10-year review are relatively low-throughput [89]. The protocol used here required about 1 hour to enrich ~9.5 mL of blood (200 µL/min) for the gene expression assay and longer for imaging. The extended processing time may limit clinical utilization, but the automated system limits hands-on time. Although filtration devices may be susceptible to clogging, the enhanced elongated pores in the MCA design allow continuous flow after CTC capture to reduce clogging [14]. Furthermore, the extended dwell time may expose CTCs and CTC clusters to extended shear stress that can damage CTC and disaggregate CTC clusters [90]. Although the pressure drop is attenuated by the design of the MCA (no pubmed citation), it is possible CTC cluster recovery was suboptimal. However, the MCA system has been shown to isolate significantly more clusters than the CELLSEARCH [91].

Overall, this study suggests that CTC gene expression and enumeration by imaging are complementary. We have also shown that the enumeration of epithelial cells is less prognostic while patients are in therapy. We speculate that this may be due to cytotoxic and cytostatic therapies that alter the morphology of CTCs resistant to therapy and make the cells more difficult to enumerate. Interestingly, this plasticity can be observed in multiplexed gene expression. As such, the detection of CTCs with EMT characteristics was highly prognostic in MBC. Further studies exploring multidimensional data including digital pathology that can recognize complicated and subtle phenotypes are needed.

## 5. Conclusions

This study reaffirmed the cut-off of ≥5 CTCs for inferior prognosis of patients with MBC using a technology that enriched epithelial and mesenchymal phenotypes. Epithelial CTC counts were prognostic before initiation of therapy and early in therapy, whereas a shift towards mesenchymal CTC phenotypes as detected by gene expression was associated with disease progression. Discordances between CTCs and tissue biopsy, as seen here in patients with HER2+ CTCs and EGFR+ CTCs, offer opportunities to explore alternative therapies as CTCs better represent the metastatic scenario than tumor tissue. Overall, this study suggests that with the use of a multimodal liquid biopsy and a protein-agnostic enrichment platform, enumeration and gene expression profiling of CTCs are complementary, offering a broader, more readily accessible picture of tumor response to therapy.

## Figures and Tables

**Table 1:** Summary of demographics and clinical characteristics of patients with MBC that participated in the study.

**Cohort**	Number of Patients	184
	Number of samples	755
	Number of healthy volunteers	57
	Number of healthy volunteer samples	118
**Patient Age**	<35	15 (8.2%)
	35–49	57 (31.0%)
	50–65	75 (40.8%)
	>65	37 (20.1%)
**Met** **Histologic Subtype**	HR+ HER2−	106 (57.6%)
	HR− HER2+	10 (5.4%)
	HR+ HER2+	14 (7.6%)
	TNBC	37 (20.1%)
	NA *	17 (9.2%)
**IBC**	IBC	23 (12.5%)
	Non-IBC	161 (87.5%)
**Lines of Treatment at Enrollment**	1	140 (76.1%)
	>1	44 (23.9%)
**De novo Stage IV**	De novo Stage IV	45 (24.5%)
	Previous DX	139 (75.5%)
**Status**	Alive	104 (56.5%)
	NED	14 (7.6%)
	Progressed	105 (57.1%)
	Deceased	80 (43.5%)
**Race**	White	137 (74.5%)
	Hispanic	10 (5.4%)
	Black	21 (11.4%)
	Asian	7 (3.8%)
	Other	9 (4.9%)
**Clinical Benefit of Met therapy** **Best Response**	Yes (CR + PR + SD)	115 (62.5%)
	No (PD)	43 (23.4%)
	NA	26 (14.1%)

* HER2+HR unknown is classified as NA. CR, complete response DX, diagnosis HR, hormone receptor (ER or PR) IBC, inflammatory breast cancer NA, not available NED, no evidence of disease PD, progressive disease PR, partial response SD, stable disease TNBC, triple negative (PR, HER 2).

**Table 2:** Time-dependent Multivariate Analysis.

		PFS		OS	
Covariate	Level	HR (95% CI)	*p*-Value	HR (95% CI)	*p*-Value
CTC count	<5	1.000		1	
	≥5	2.251 (1.628–3.113)	<0.0001	4.894 (2.854–8.393)	<0.0001
HR/HER2	HR+/HER2+	1.000		1	
(metastatic site)	HR+/HER2−	0.717 (0.409–1.258)	0.1968	0.482 (0.115–2.024)	0.3191
	HR−/HER2+	1.057 (0.571–1.959)	0.859	0.893 (0.267–2.993)	0.8549
	HR−/HER2−	1.725 (1.267–2.348)	0.0005	3.389 (1.973–5.820)	<0.0001
FGFR1	Low	1			
	High	1.470 (1.074–2.012)	0.0138	-	-

CTC, circulating tumor cell; HR, hormone receptor (ER or PR); IBC, inflammatory breast cancer; NA, not available; NED, no evidence of disease; PD, progressive disease; PR, partial response; SD, stable disease; TNBC, triple negative (ER, PR, HER2).

## Data Availability

The data that support the findings of this study are available from the corresponding authors upon request.

## Supplementary Materials

The following supporting information can be downloaded at: https://www.mdpi.com/article/10.3390/cancers15051616/s1, Figure S1. Study design; Figure S2. Distribution of CTC Counts; Figure S3. CTC Count Distribution; Figure S4. Progression-free survival across CTC cut-offs at baseline and all time points combined; Figure S5. Multivariate Analysis including CTC counts; Figure S6. Dynamic change in CTC counts (PFS and OS); Figure S7. Progressors have higher expression than non-progressors on-therapy; Figure S8. Select genes expressed higher in progressors; Figure S9. Change in gene expression from baseline; Figure S10. Select genes decrease with time in non-progressors; Figure S11. ≥4 Positive genes are prognostic; Figure S12. Mean Cumulative Function for progression events; Figure S13. Genes are prognostic on-therapy in multivariate analysis; Figure S14. ERBB2+ CTC are Prognostic in HER2- Met Tumors; Figure S15. A case study of a metastatic Inflammatory Breast Cancer patient; Figure S16. A case study of a metastatic Inflammatory Breast Cancer patient; Table S1. Genes included in the study; Table S2. Time Dependent Effects. Time Dependent Effects (PFS based on Prentice Williams and Peterson counting process model (PWP-CP); Table S3. Mixed Effect Model on clinical benefit: Mixed effect model: With or without time effect was considered.
