# Supplementary material for: Phenotypic Plasticity in Circulating Tumor Cells Is Associated with Poor Response to Therapy in Metastatic Breast Cancer Patients
Source: Cancers (Basel). 2023 Mar 6;15(5):1616. doi: 10.3390/cancers15051616 (PMC10000974; doi:10.3390/cancers15051616)
Supplement: Supplementary file 1 [file cancers-15-01616-s001.zip › cancers-2244739-supplementary.pdf]

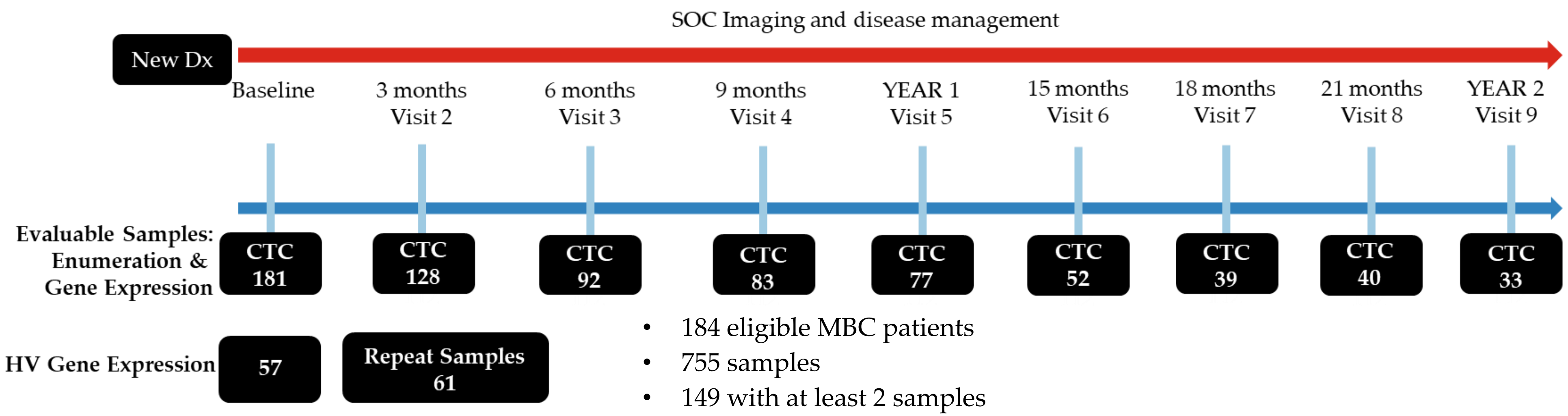

Figure S1. Study design of the clinical study PA16-0507 for the longitudinal sampling of blood from patients with MBC.

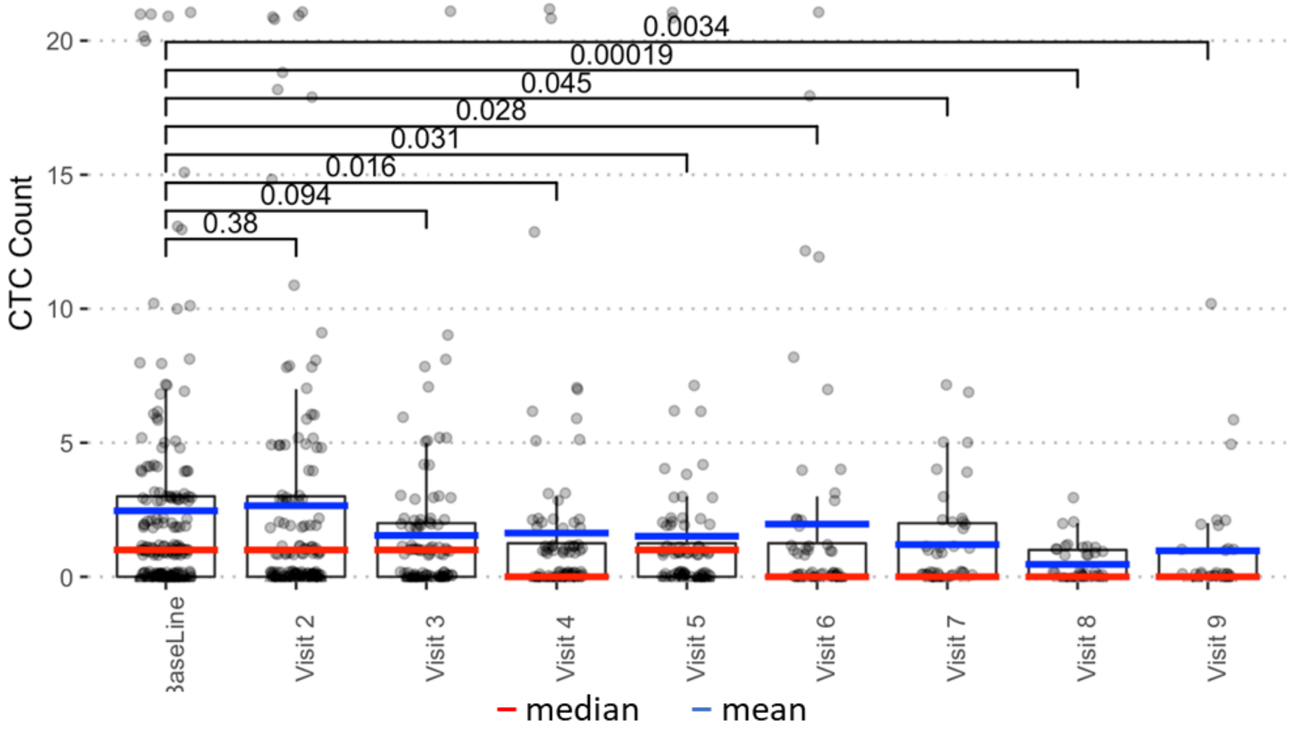

Figure S2. Distribution of CTC Counts (Wilcoxon signed-rank test with count set to 21). CTC counts of patients with MBC decrease with time on protocol.

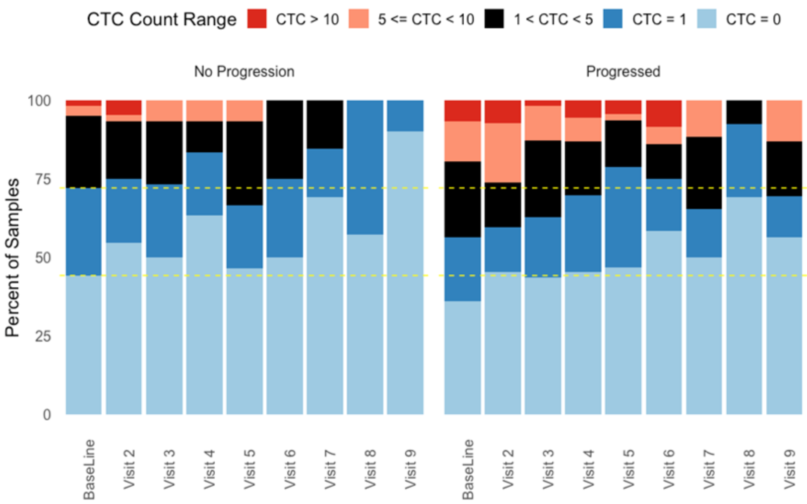

(a)

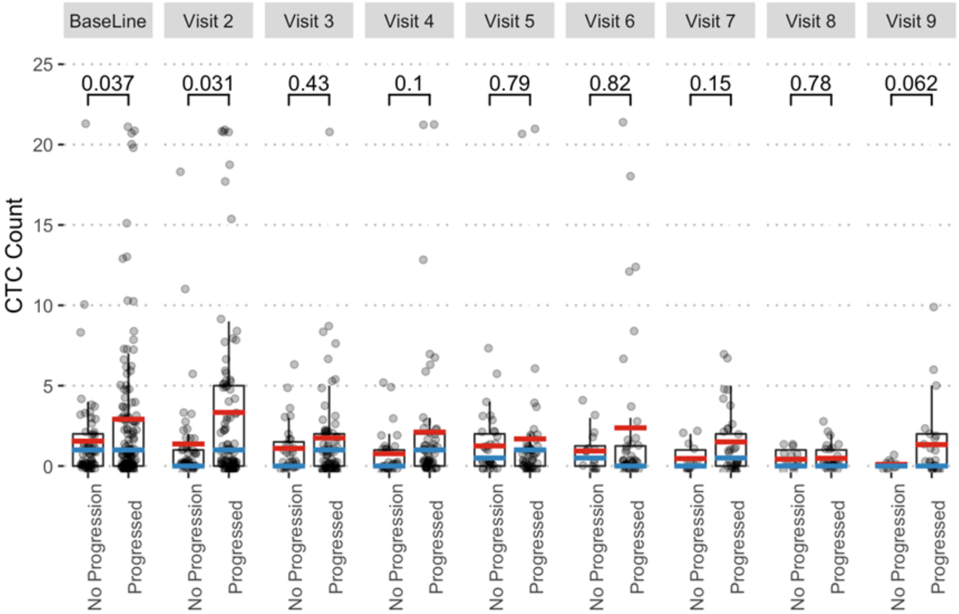

(b)

Figure S3. (a) Frequency of CTC Count classification- Progression vs NO progression. (b) t-test with CTC count set to 21. Samples devoid of observable CTCs (0 CTCs) were more frequent in patients with stable disease (lower yellow dashed line) compared to patients with progression. At later time points, survivorship bias likely influences the distribution presented. Note that each time point is shown as a percentage of samples, which places a larger weight on individual samples, as the number of patients with many repeat visits is rare.

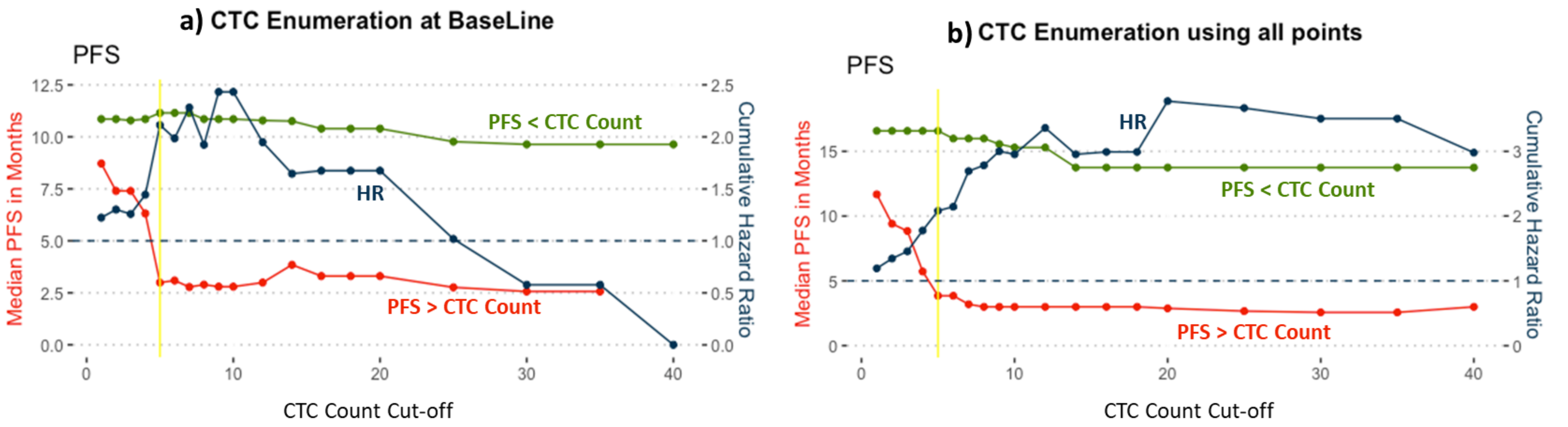

Figure S4. Progression-free survival across CTC cut-offs at baseline (a) and all time points combined (b). The rarefaction curve shows that at low CTC cutoffs, there was a small difference in PFS between patients with counts below the threshold (green) and those with counts above the threshold (red). However, as the CTC cutoff increased, PFS decreased, and the hazard ratio (HR) (blue) increased in patients with higher CTC counts. There was an inflection point in CTC-positive PFS at a cutoff of 5 or more CTCs, suggesting a threshold of 5 CTCs as the optimal cutoff to use. At baseline, the HR leveled off at the cutoff of ≥5 CTC; however, when considering all study time points, the HR continued to increase with higher cutoff values (Figure S4). Using OS had minimal utility in this analysis, as median OS was not reached for patients with CTC counts below the threshold.

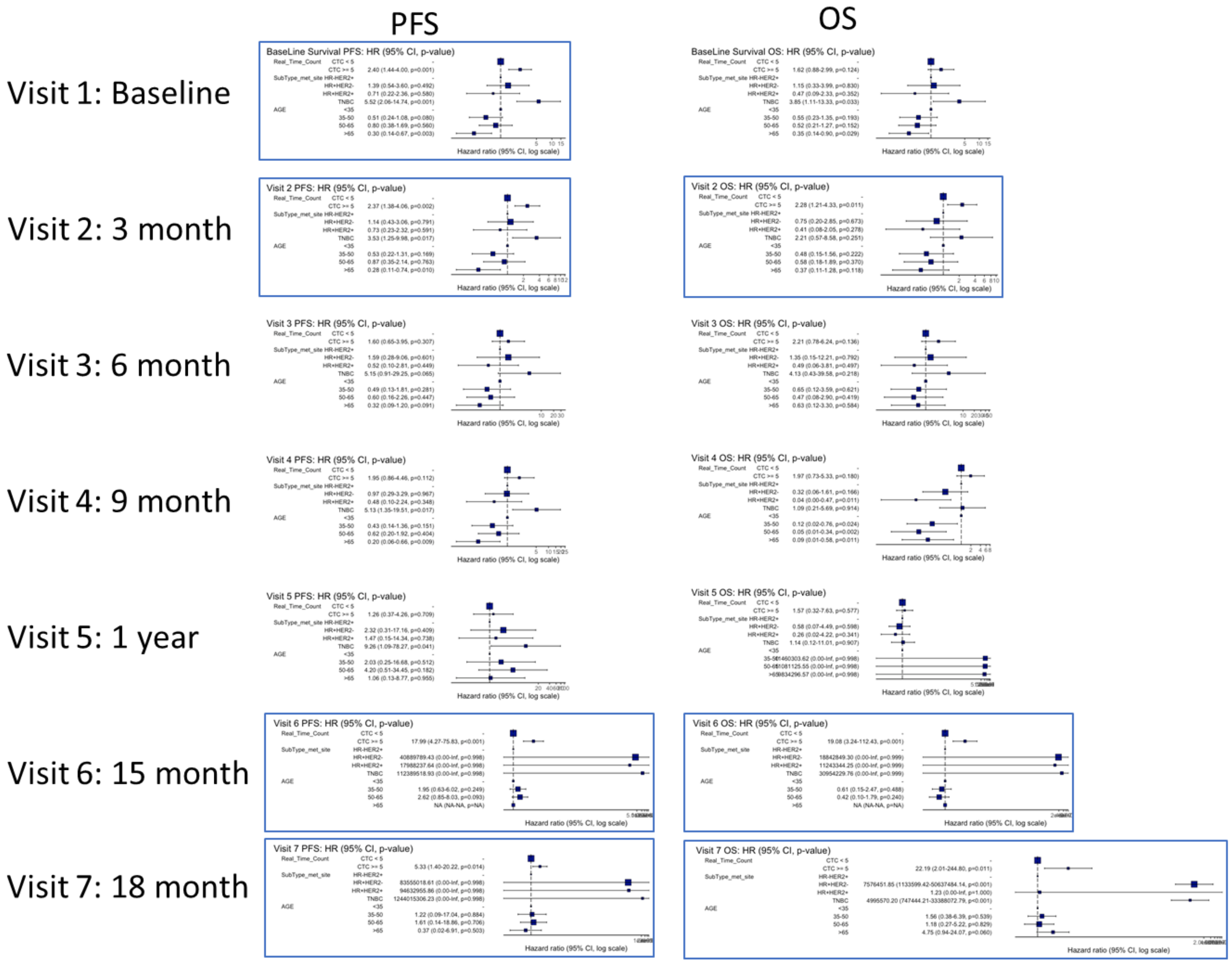

Figure S5. Multivariate Analysis including CTC counts- Multivariate analyses for PFS and OS at each timepoint including CTC Count ≥ 5, metastatic tumor tissue subtype, and age. Analyses where CTC count is independent prognostic factor are highlighted in blue.

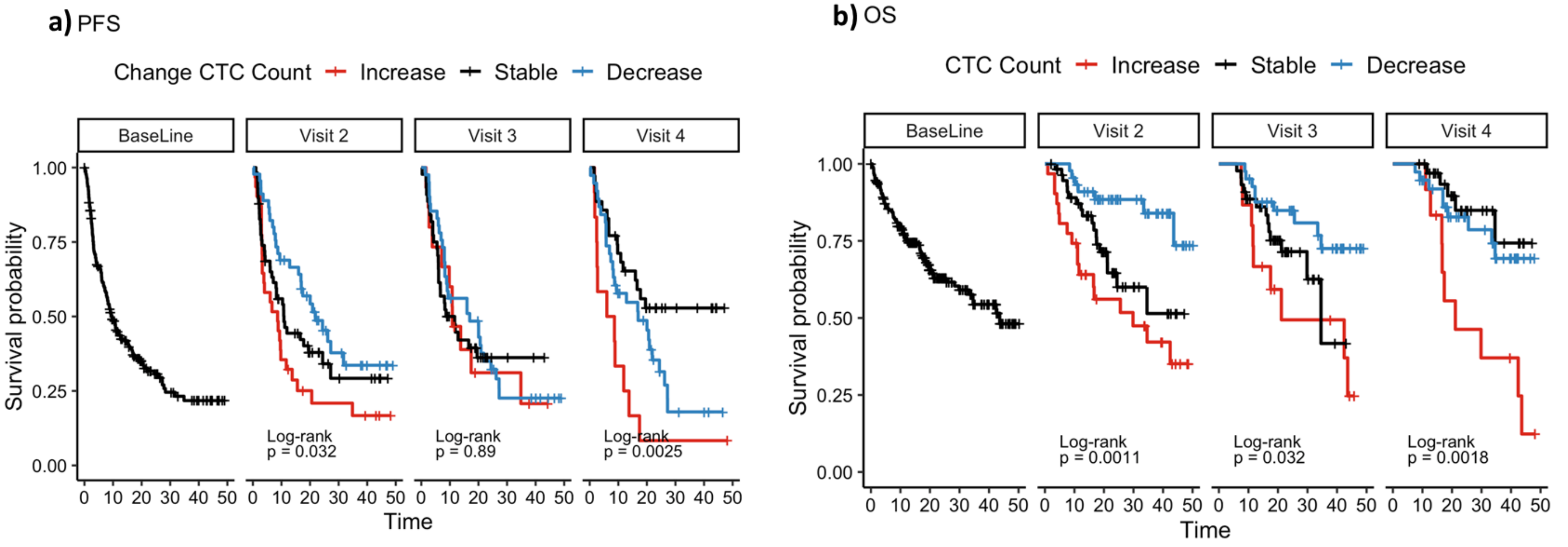

Figure S6. Dynamic change in CTC counts (PFS and OS).

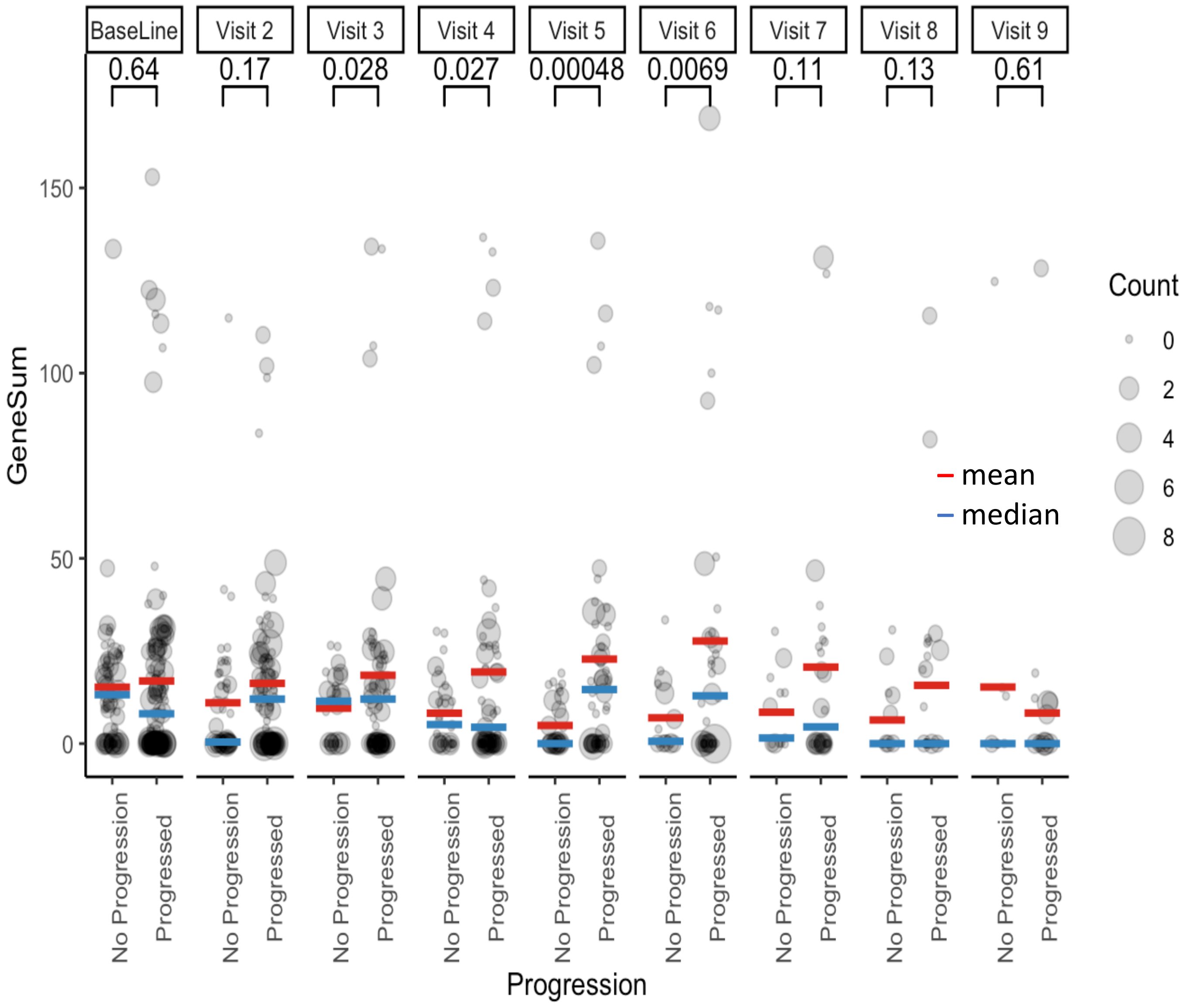

Figure S7. Progressors have higher expression than non-progressors on-therapy.

Table S1. Genes included in the study.

	**Housekeeping Genes**
**B2M:**	Beta-2-Microglobulin (Beta Chain of MHC Class I Molecules) (qHsaCID0015347)
GAPDH:	Glyceraldehyde 3-phosphate dehydrogenase (qHsaCED0038674)
HPRT:	Hypoxanthine guanine phosphoribosyl transferase (qHsaCID0016375)
PTPRC/CD45:	Protein Tyrosine Phosphatase, Receptor Type C (white blood cell control) (qHsaCED0038908)
GYPA:	Glycophorin A (a red blood cell control gene) (qHsaCID0010750)
	**Epithelial-related genes**
CDH1:	Cadherin 1 (Epithelial (E)-Cadherin) (qHsaCID0015365)
EGFR:	Epidermal Growth Factor Receptor (qHsaCID0007564)
EPCAM:	Epithelial Cell Adhesion Molecule (qHsaCED0043827)
KRT7:	Keratin 7 (basic, low molecular weight cytokeratin) (qHsaCED0038533)
KRT18:	Keratin 18 (acidic, low molecular weight cytokeratin) (qHsaCED0035037)
MUC1:	Mucin 1 (Cell Surface Associated) (qHsaCED0019841)
	**Mesenchymal and EMT-related genes**
AXL:	AXL Tyrosine-protein kinase receptor (qHsaCID0008470)
CDH2:	Cadherin 2 (N-Cadherin) (qHsaCIP0029771)
FN1:	Fibronectin 1 (qHsaCID0012349)
SNAI2:	Snail Family Transcriptional Repressor 2 (SLUG) (qHsaCID0011342)
ZEB2:	Zinc Finger E-Box Binding Homeobox 2 (qCfaCEP0009109)
	**Other cancer-related genes**
ALDH1A1:	Aldehyde Dehydrogenase 1 Family Member A1 (qHsaCID0018574)
BCL2:	B-cell lymphoma 2 (qHsaCED0057245)
CD274/PD-L1:	Programmed Cell Death 1 Ligand 1(qHsaCID0036468)
ERBB2/HER2:	Receptor Tyrosine Kinase 2 (Human Epidermal Growth Factor Receptor 2) (qHsaCED0045039)
FGFR1:	Fibroblast Growth Factor Receptor 1 (qHsaCED0042405)
MET:	Tyrosine-protein kinase Met/hepatocyte growth factor receptor (HGFR) (qHsaCED0002004)

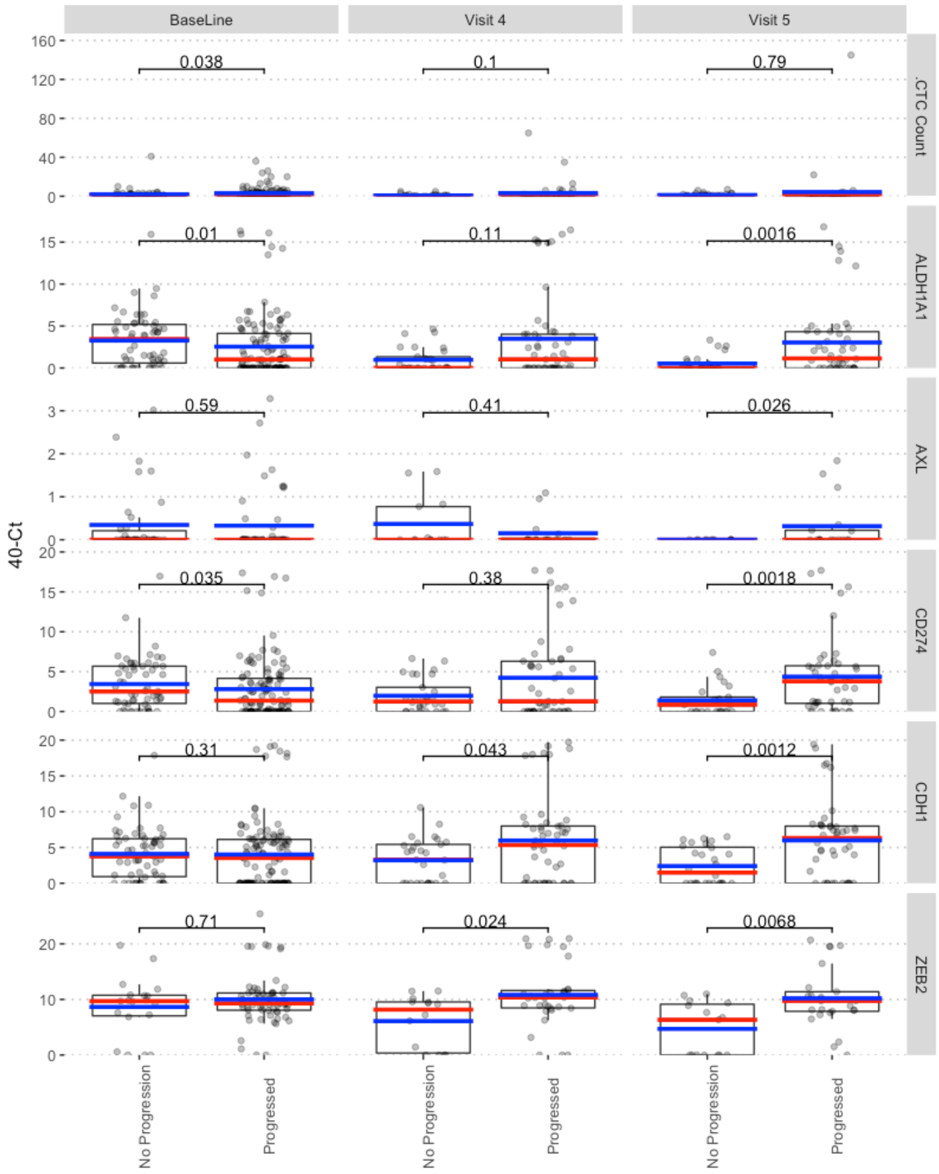

Figure S8. Select genes expressed higher in progressors (Wilcoxon rank-sum Test)

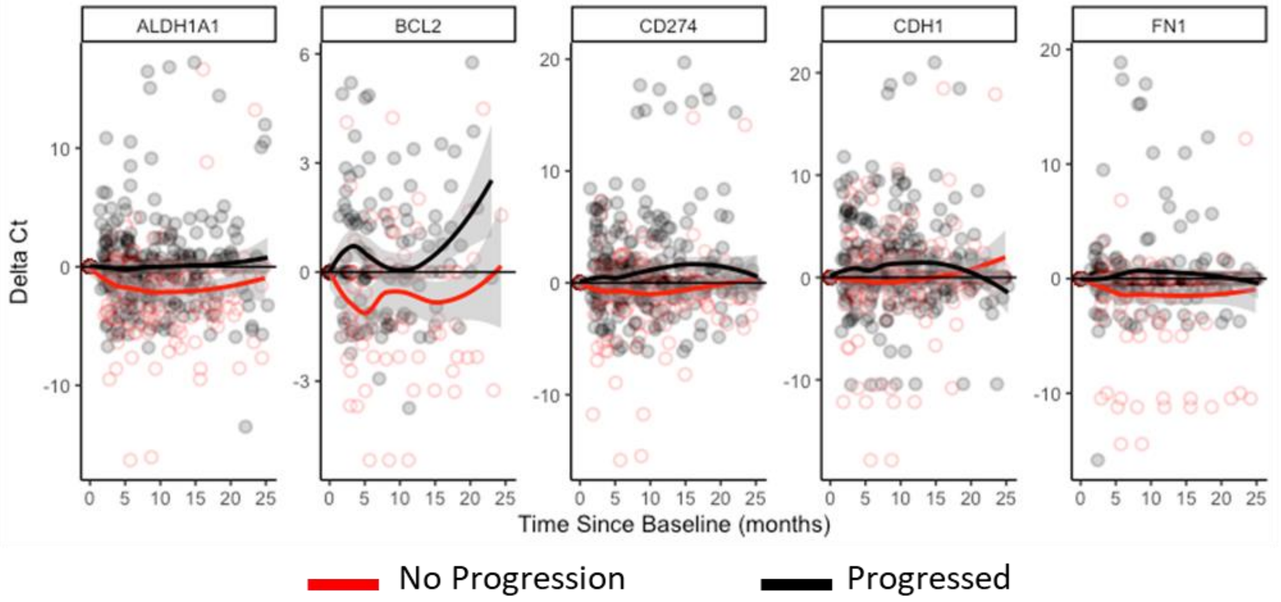

Figure S9. Select gene expression is high in progressors- Change in gene expression from baseline

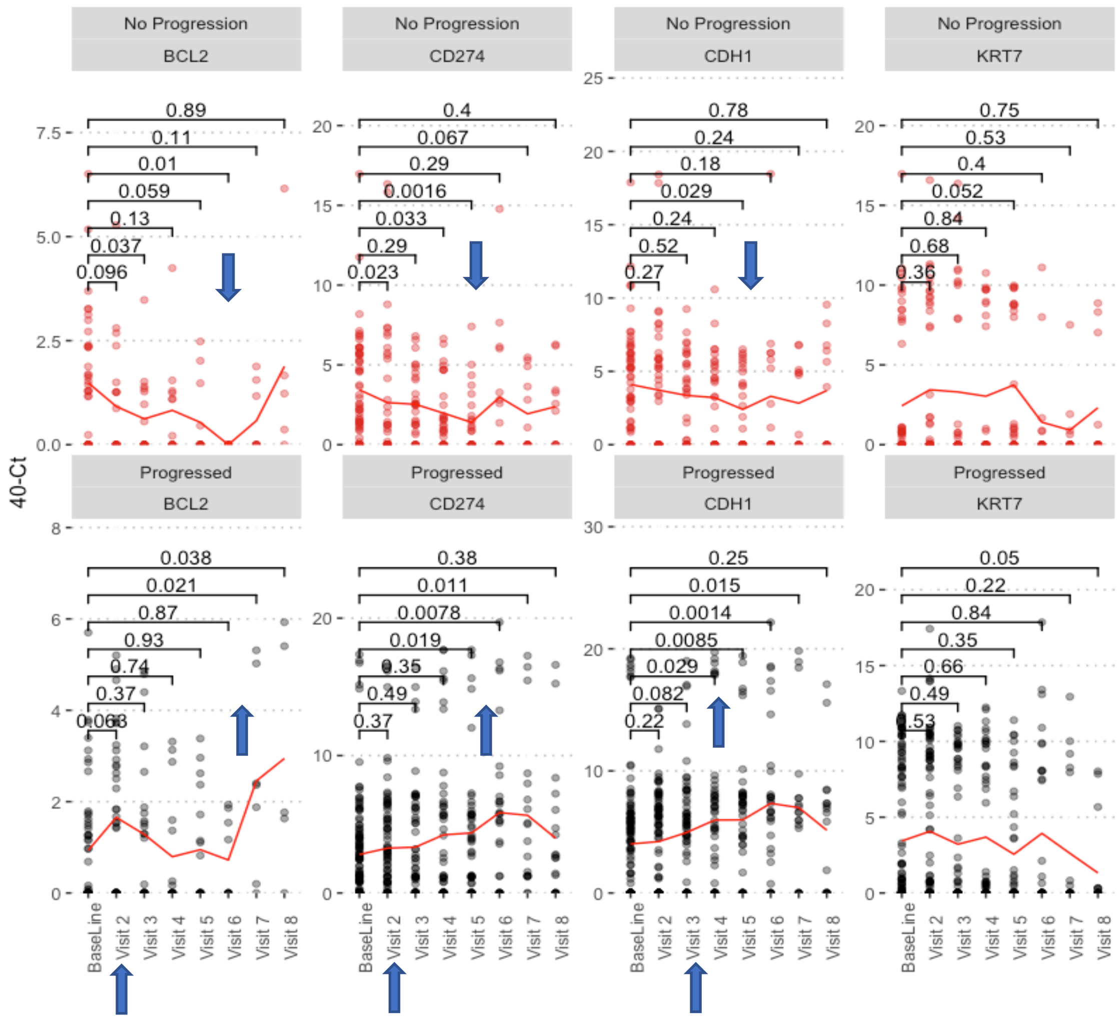

Figure S10. Select genes decrease with time in non-progressors.

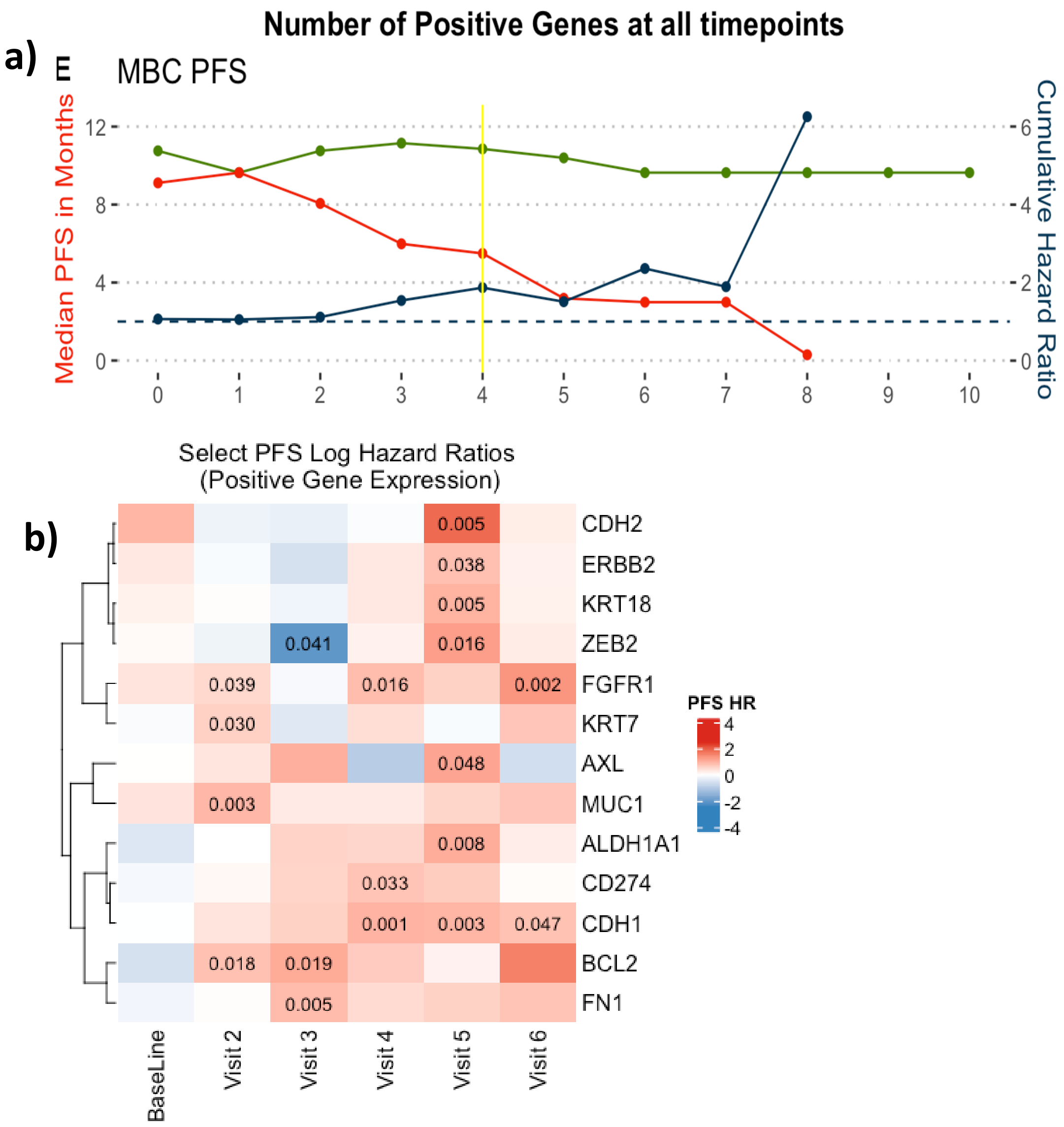

Figure S11. ≥4 Positive genes are prognostic .

Cox proportional hazard modeling was used to determine the effect of positive expression of individual genes on the time to first progression. As with the number of CTCs, PFS decreased with increasing numbers of positive CTC genes, but there was not a clear cut-point. The HR leveled off at ≥4 positive genes, and the number of samples with ≥5 positive genes started to drop dramatically, so a cutoff of ≥4 positive CTC genes was chosen for stratification.

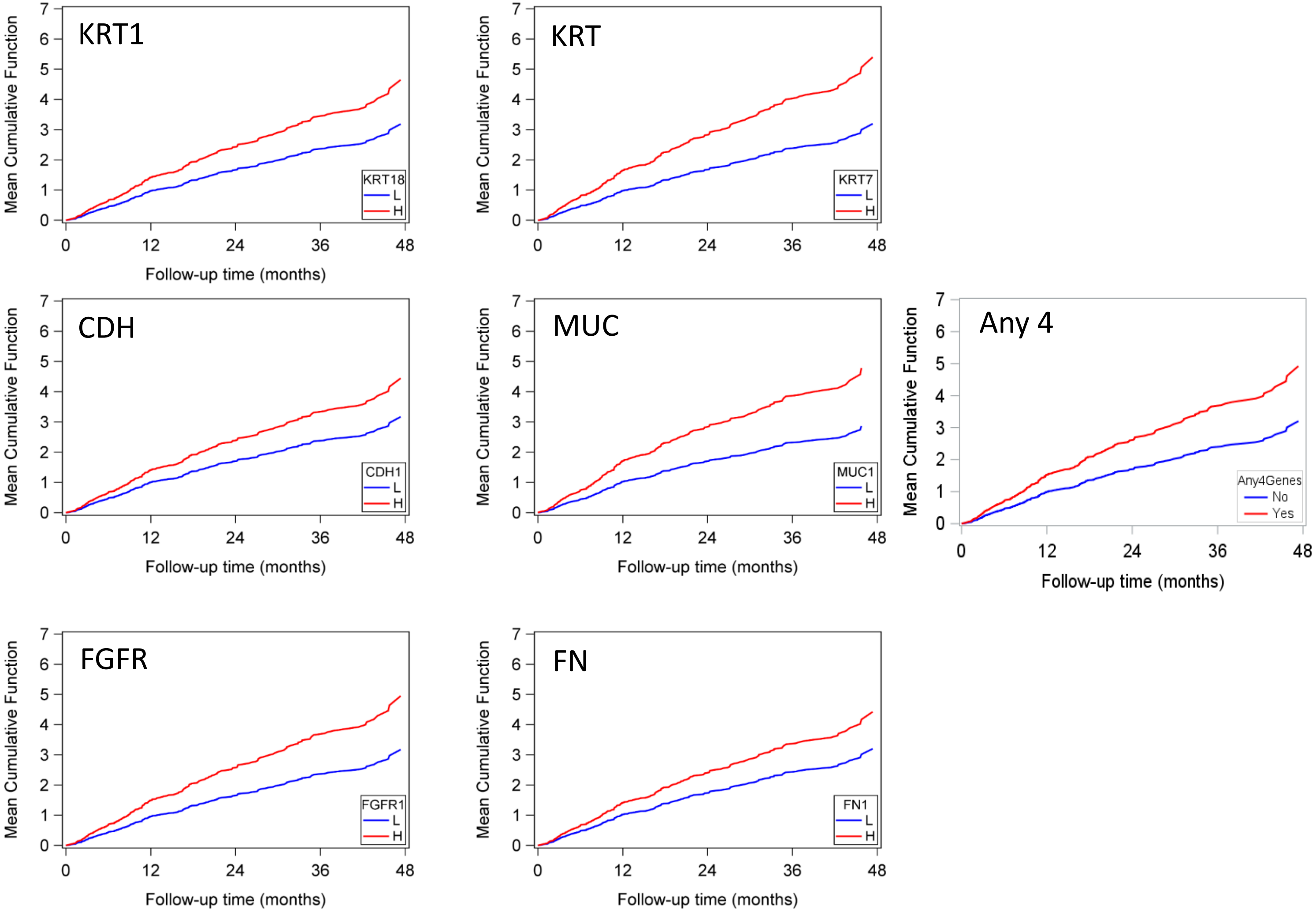

Figure S12. Mean Cumulative Function for progression events.

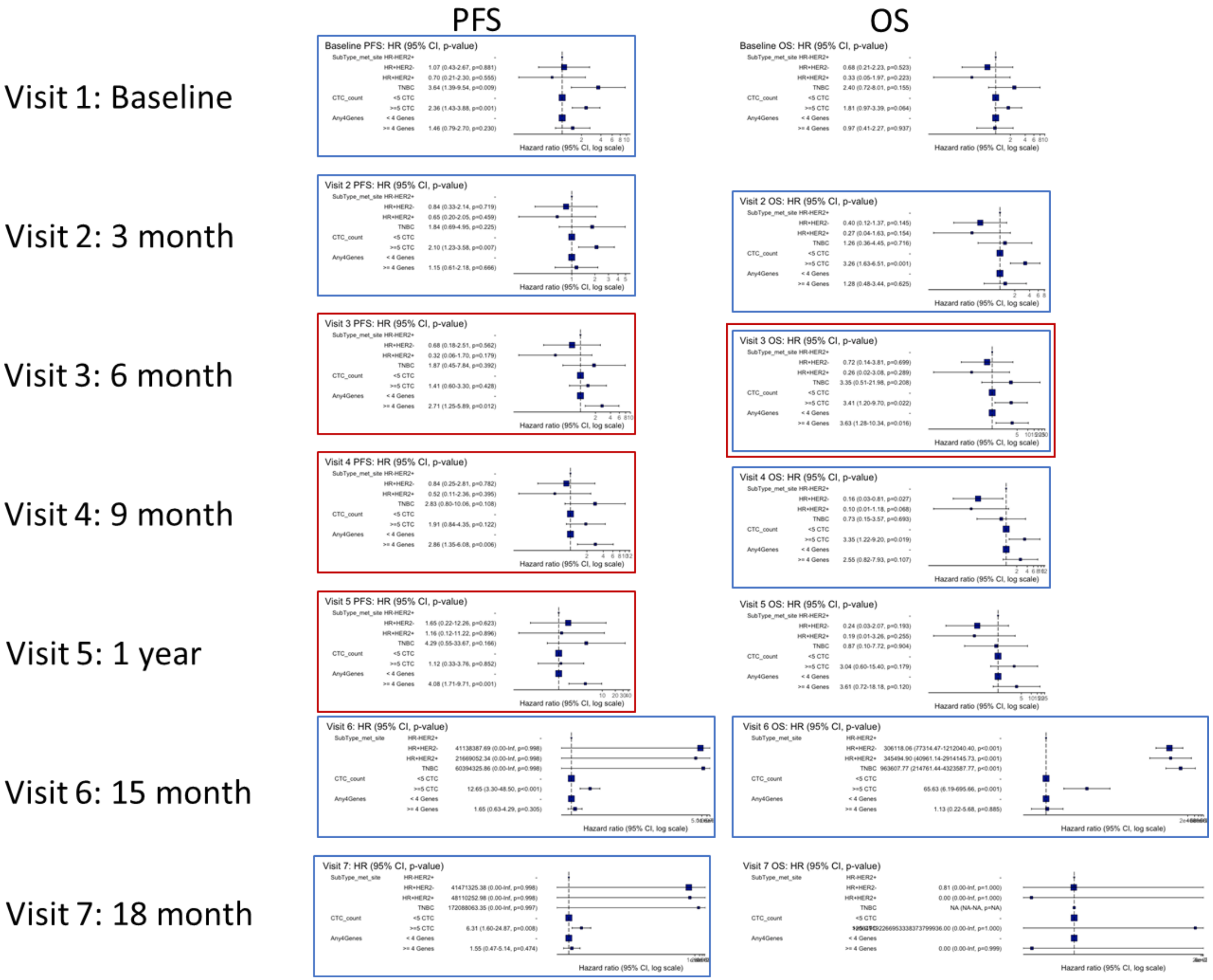

Figure S13. Genes are prognostic on-therapy in multivariate analysis. Multivariate analyses at each time point including metastatic tumor tissue subtype, CTC Count ≥ 5 and at detection of least 4 CTC genes. Analyses where CTC count is independent prognostic factor are highlighted in blue and analyses where gene expression is independent prognostic factor are highlighted in red. .

Table S2. Time Dependent Effects. Time Dependent Effects (PFS based on Prentice Williams and Peterson counting process model (PWP-CP).

		**Univariate**				**Multivariate**			
Covariate	Level	PFS HR (95% CI)	P-value	OS HR (95% CI)	P-value	PFS HR (95% CI)	P-value	OS HR (95% CI)	P-value
Age	In 1 Unit Change	0.995 (0.986-1.005)	0.3261	1.000 (0.982-1.018)	0.961				
Lines of Tx	In 1 Unit Change	1.281 (1.041-1.577)	0.0191	1.506 (1.019-2.226)	0.04				
Primary HR/HER2	HR+/HER2+	1		1					
	HR+/HER2-	0.698 (0.459-1.062)	0.0933	0.269 (0.064-1.120)	0.0711				
	HR-/HER2+	0.759 (0.388-1.483)	0.4193	0.726 (0.174-3.031)	0.6607				
	HR-/HER2-	1.416 (0.971-2.063)	0.0706	2.455 (1.415-4.258)	0.0014				
Metastatic HR/HER2	HR+/HER2+	1		1		1		1	
	HR+/HER2-	0.779 (0.432-1.404)	0.4058	0.438 (0.104-1.834)	0.2584	0.717 (0.409-1.258)	0.2458	0.482 (0.115-2.024)	0.3191
	HR-/HER2+	1.062 (0.542-2.078)	0.8615	1.293 (0.394-4.248)	0.6718	1.057 (0.571-1.959)	0.8590	0.893 (0.267-2.993)	0.8549
	HR-/HER2-	1.625 (1.158-2.279)	0.0049	3.486 (2.030-5.987)	<0.0001	1.725 (1.267-2.348)	0.0005	3.389 (1.973-5.820)	<.00001
CTC Count	>=5	2.104 (1.623-2.726)	<0.0001	4.827 (2.909-8.009)	<0.0001	2.251 (1.628-3.113)	<0.0001	4.894 (2.854-8.393)	<0.0001
Sum CTC gene levels	In 1 Unit Change	1.002 (0.999-1.006)	0.1965	1.003 (0.995-1.010)	0.4746				
Any positive CTC genes	Yes	1.220 (0.916-1.624)	0.1731	1.074 (0.621-1.857)	0.7987				
Any 4 CTC Genes*	Yes	1.274 (0.950-1.709)	0.1064	1.957 (1.087-3.526)	0.0253				
ALDH1A1	High	1.102 (0.816-1.489)	0.5250	1.080 (0.574-2.031)	0.8122				
AXL	High	0.893 (0.540-1.476)	0.6590	0.693 (0.205-2.347)	0.5561				
BCL2	High	1.290 (0.835-1.993)	0.2511	1.009 (0.399-2.554)	0.9844				
CD274	High	1.111 (0.811-1.523)	0.5113	1.413 (0.751-2.657)	0.2838				
CDH1	High	1.224 (0.925-1.619)	0.1582	1.296 (0.713-2.355)	0.3947				
CDH2	High	0.991 (0.533-1.844)	0.9778	0.736 (0.176-3.078)	0.675				
EGFR	High	1.235 (0.804-1.898)	0.3343	1.198 (0.516-2.782)	0.6739				
EPCAM	High	0.987 (0.750-1.299)	0.9245	1.090 (0.589-2.016)	0.7848				
ERBB2	High	1.298 (0.934-1.803)	0.1202	1.486 (0.750-2.941)	0.256				
FGFR1	High	1.372 (1.033-1.822)	0.0288	1.692 (0.949-3.014)	0.0744	1.470 (1.074-2.012)	0.0138		
FN1	High	1.228 (0.894-1.689)	0.2050	1.795 (1.002-3.218)	0.0494				
FOXC2	High	0.773 (0.494-1.212)	0.2618	0.927 (0.381-2.258)	0.8681				
KRT7	High	1.451 (1.066-1.974)	0.0179	1.881 (0.960-3.683)	0.0654				
KRT18	High	1.270 (0.974-1.657)	0.0773	1.464 (0.847-2.528)	0.1719				
MET	High	0.743 (0.432-1.278)	0.2832	0.760 (0.259-2.234)	0.6182				
MUC1	High	1.409 (1.056-1.880)	0.0198	2.118 (1.161-3.862)	0.0144				
SNAI2	High	1.042 (0.757-1.435)	0.8000	0.778 (0.335-1.806)	0.5592				
ZEB2	High	0.976 (0.637-1.496)	0.9106	0.825 (0.293-2.326)	0.7159				

*If at least 4 of the following genes are positive (BCL2, CD274, CDH1, EPCAM, FGFR1, FN1, KRT18, MET, MUC1).

Table S3. Mixed Effect Model on clinical benefit: Mixed effect model: With or without time effect was considered; subject covariance type of variance component (VC) structure, compound symmetry (CS) structure and AR(1) (first-order Autoregressive structure) structure were considered; a model with the smallest AIC was selected.

**Variable**	**AIC**	**Estimate of clinical benefit=Yes vs. No**	**P-value**	**Significant criterion by Bonferroni-Holm correction**	**Significant**
FN1	533.28	-0.15837	**0.00001**	0.05/(43-1+1)=0.00116	**Yes**
MUC1	416.21	-0.13697	**0.00052**	0.05/(43-2+1)=0.00119	**Yes**
Standardized EPCAM	2049.14	-0.56612	**0.00064**	0.05/(43-3+1)=0.00122	**Yes**
Standardized KRT18	2259.14	-0.50897	**0.00263**	0.05/(43-4+1)=0.00125	
HPRT1	573.3	-0.15613	**0.00448**	0.05/(43-5+1)=0.00128	
Standardized FGFR1	2644.66	-0.60398	**0.00688**	0.05/(43-6+1)=0.00132	
Standardized CDH2	1339.03	0.53932	**0.02304**	0.05/(43-7+1)=0.00135	
Standardized MUC1	2517.85	-0.75359	**0.0235**	0.05/(43-8+1)=0.00139	
FGFR1	624.44	-0.10779	**0.0338**	0.05/(43-9+1)=0.00143	
Standardized ERBB2	1888.46	-0.2725	**0.03949**	0.05/(43-10+1)=0.00147	
KRT7	340.53	-0.08926	**0.04016**	0.05/(43-11+1)=0.00152	
BCL2	319.77	-0.12121	0.05142	0.05/(43-12+1)=0.00156	
Standardized KRT7	2160.64	-0.26587	0.0672	0.05/(43-13+1)=0.00161	
Standardized CDH1	1783.25	-0.19104	0.06982	0.05/(43-14+1)=0.00167	
CTC Count	3981.72	-1.3451	0.13178	0.05/(43-18+1)=0.00192	

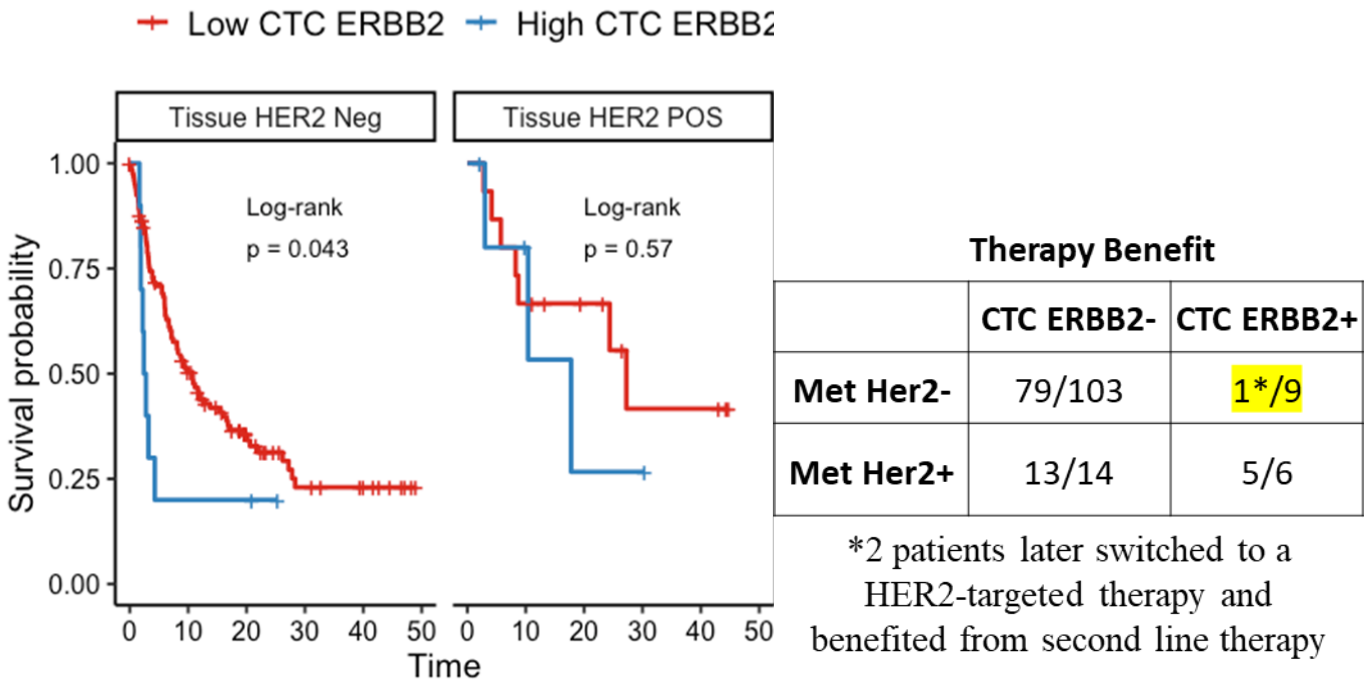

Therapy Benefit

	CTC ERBB2-	CTC ERBB2+
**Met Her2-**	79/103	1*/9
**Met Her2+**	13/14	5/6

*2 patients later switched to a HER2-targeted therapy and benefited from second line therapy

Figure S14. ERBB2+ CTC are Prognostic in HER2- Met Tumors

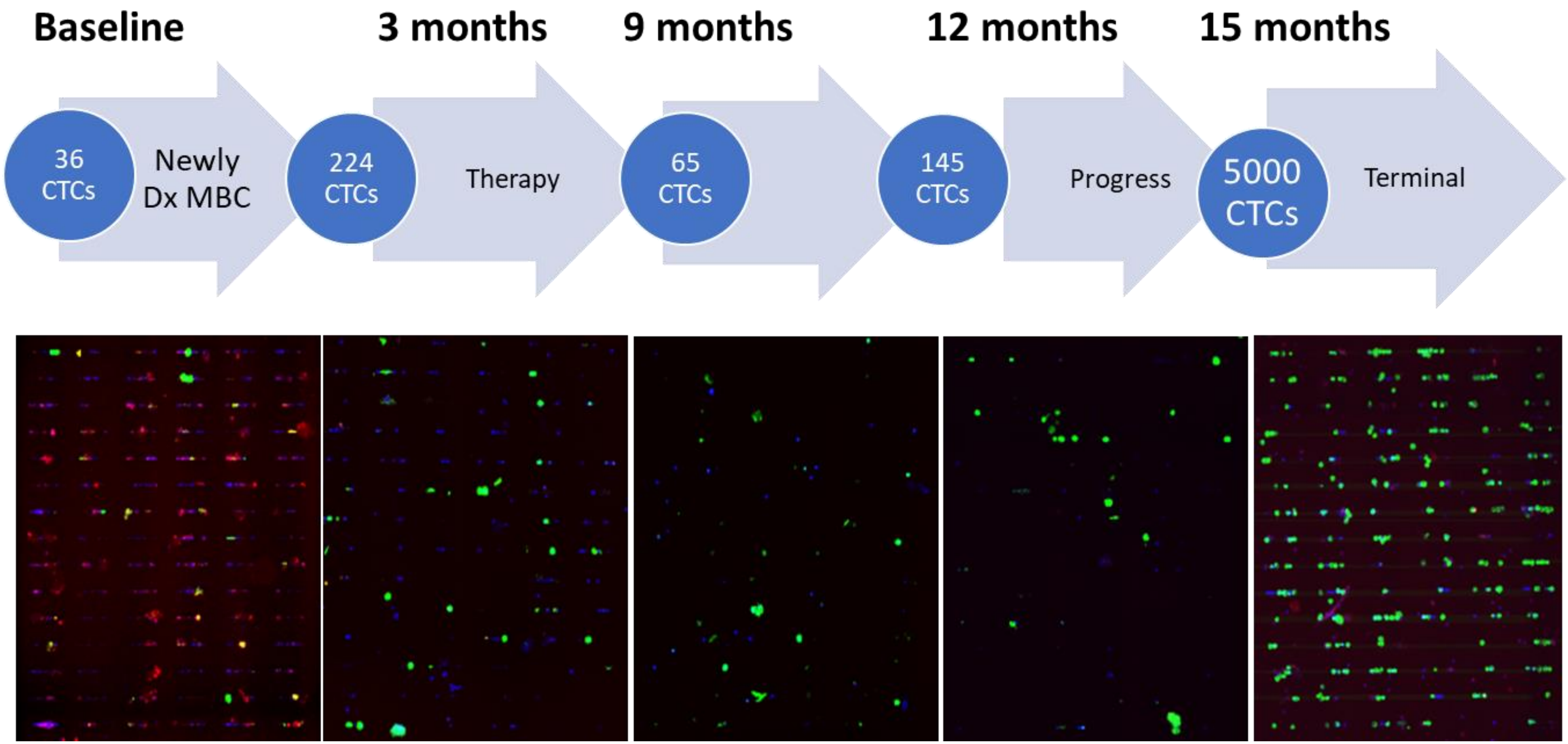

Figure S15. A case study of a metastatic Inflammatory Breast Cancer patient. The case study of a 55-year-old female with recurrent MBC (ER, PR: positive, HER2: negative) is shown (Figure S15). The patient enrolled with newly diagnosed metastatic disease, at which time 36 CTCs were counted in the blood sample. CTC counts remained high during therapy but dropped with second-line therapy and palliative radiation. A new liver metastatic site was diagnosed at 12 months concurrent with a rebound in CTC count to 145. Subsequently, the CTC count spiked to about 5000, and the patient died shortly thereafter. .

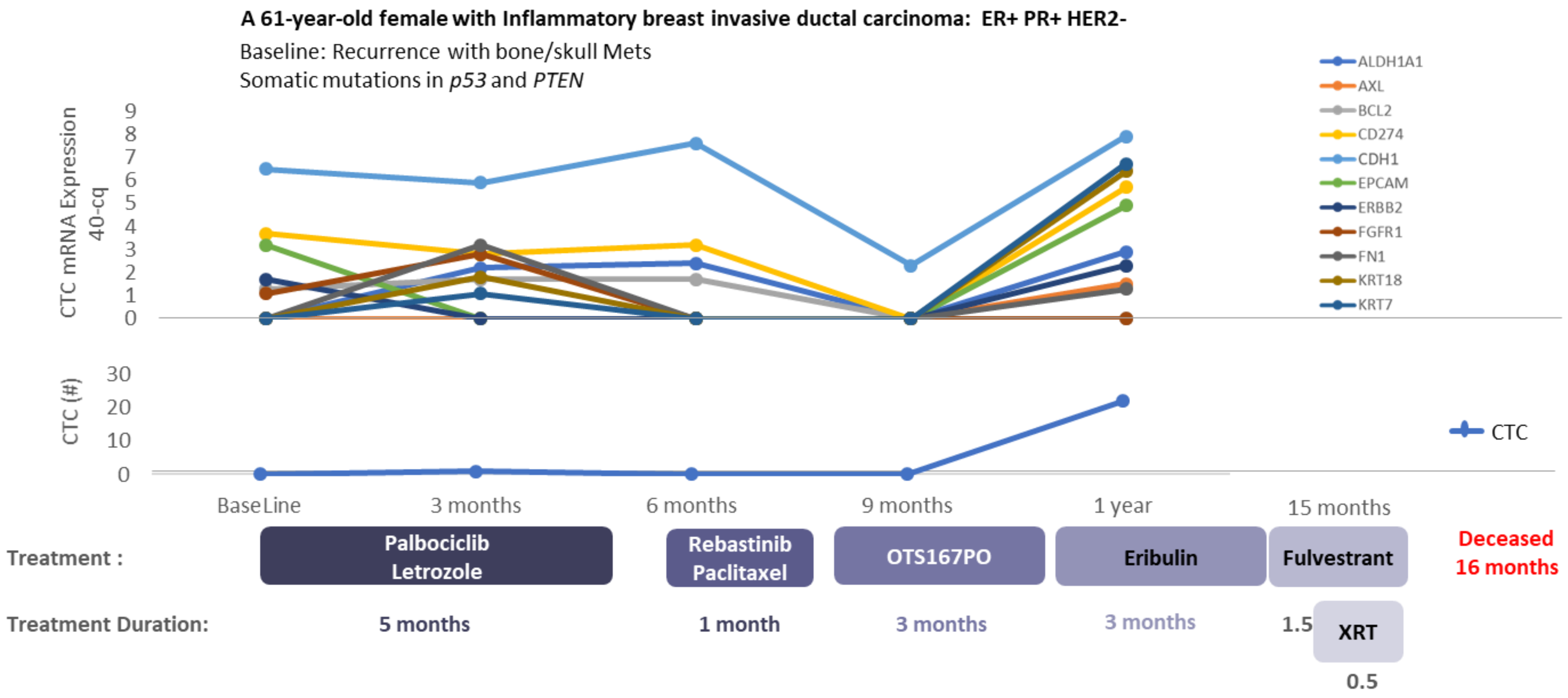

Figure S16. A case study of a metastatic Inflammatory Breast Cancer patient. A second case study shows a 61-year-old female with inflammatory breast cancer with recurrent metastasis presenting with 0 CTCs at baseline. Multiple treatment modalities were associated with dynamic changes in gene expression levels. Her CTC counts spiked a year later along with a surge in CTC-related gene expression before her death.
